# Culturing and Molecular Approaches for Identifying Microbiota Taxa Impacting Children’s Obesogenic Phenotypes Related to Xenobiotic Dietary Exposure

**DOI:** 10.3390/nu14020241

**Published:** 2022-01-06

**Authors:** Ana López-Moreno, Ángel Ruiz-Moreno, Jesús Pardo-Cacho, Klara Cerk, Alfonso Torres-Sánchez, Pilar Ortiz, Marina Úbeda, Margarita Aguilera

**Affiliations:** 1 Department of Microbiology, Faculty of Pharmacy, University of Granada, 18071 Granada, Spain; angel173@correo.ugr.es (Á.R.-M.); jesusparugr99@gmail.com (J.P.-C.); klaracerk@ugr.es (K.C.); alfonsotorress@correo.ugr.es (A.T.-S.); piortiz@ugr.es (P.O.); marinaubeda@correo.ugr.es (M.Ú.); 2 Center of Biomedical Research, Institute of Nutrition and Food Technology “José Mataix”, University of Granada, 18016 Granada, Spain; 3 Microbiota Laboratory, IBS: Instituto de Investigación Biosanitaria ibs, 18012 Granada, Spain

**Keywords:** culturomics, bioinformatics, obesogens, BPA, obesity, endocrine disruptors

## Abstract

Integrated data from molecular and improved culturomics studies might offer holistic insights on gut microbiome dysbiosis triggered by xenobiotics, such as obesity and metabolic disorders. Bisphenol A (BPA), a dietary xenobiotic obesogen, was chosen for a directed culturing approach using microbiota specimens from 46 children with obesity and normal-weight profiles. In parallel, a complementary molecular analysis was carried out to estimate the BPA metabolising capacities. Firstly, catalogues of 237 BPA directed-cultured microorganisms were isolated using five selected media and several BPA treatments and conditions. Taxa from Firmicutes, Proteobacteria, and Actinobacteria were the most abundant in normal-weight and overweight/obese children, with species belonging to the genera *Enterococcus*, *Escherichia*, *Staphylococcus*, *Bacillus*, and *Clostridium*. Secondly, the representative isolated taxa from normal-weight vs. overweight/obese were grouped as BPA biodegrader, tolerant, or resistant bacteria, according to the presence of genes encoding BPA enzymes in their whole genome sequences. Remarkably, the presence of sporobiota and concretely *Bacillus* spp. showed the higher BPA biodegradation potential in overweight/obese group compared to normal-weight, which could drive a relevant role in obesity and metabolic dysbiosis triggered by these xenobiotics.

## 1. Introduction

Exposure to obesogens and endocrine disrupting chemicals (EDCs) can lead to microbial and molecular dysbiosis [1,2], which is based on misbalanced taxa compositions and associated with several metabolic diseases, such as type 2 diabetes, obesity, and other endocrine disorders [3,4,5]. EDCs are also considered microbiota disrupting chemicals (MDCs) [6]. Concretely, Bisphenol A (BPA), as one of the more representative, studied, and controversial EDCs, is widely used in polycarbonate and epoxy resins and packages [7]. The contaminant is widely present in the environment, including soils, sediments, aquatic environments; and water, air, and dust particles [8]. Several routes of human exposure to BPA have been described, including the digestive system (ingestion) through exposure to food packaging, drinking containers, and dental monomers [9,10]; the vertical transmission (maternofetal) [11]; the respiratory system (inhalation) [12]; and the integumentary system (skin and eye contact) through exposure to the thermal paper of receipts, contact lenses, and feminine hygiene products [13,14]. The presence of these obesogens and MDCs in humans has been also confirmed in serum, urine, saliva, hair, tissue, and blood [15,16]. Thus, BPA removal from the natural environment is of increasing interest worldwide. Several studies have identified biological effective ways to remove it through organisms, such as bacteria, fungi, algae, and plants [17,18]. The evidence of the impact of dietary exposure to BPA has led the industry to use analogous compounds, such as bisphenol S (BPS), bisphenol F (BPF), etc. However, recent studies showed that some of these analogues may be even more harmful than BPA [19]. In the case of BPS, evidence suggests that it acts as an MDC, but research in this field has remained limited [20].

Metabolism and metabolites upon ingestion of BPA undergo rapid first-pass metabolism to BPA-glucuronide, which is biologically inert and rapidly cleared in the urine [21]. Glucuronidation is the main detoxication pathway for BPA in humans and other species, understanding the differential impact and potential health risks from early-life exposure to BPA, especially in the neo-natal period and infancy [22]. BPA glucuronide is driven by uridine diphosphate glucuronosyltransferases (UGTs) in the liver and gut. However, BPA microbial metabolisation starts to be understood under the relevance of toxicomicrobiomics recent studies [23]. In this sense, the isolation, culture, and analysis of the microbial taxa components associated with adverse functional effects, would allow a better understanding of the underlying pathophysiological mechanisms and control via administration of beneficial microbes, helping to regulate the physiological hormonal axis [24]. The application of culturomics for the human microbiome description is advancing towards more effective isolations via sophisticated culture methods of the human microbiome [25]. These methods rely on culturing of human samples with different growth media under varying conditions, along with identification of isolated bacterial colonies with matrix-assisted laser desorption/ionisation time of flight mass spectrometry (MALDI-TOF MS) and 16S rRNA gene sequencing [25,26]. Culturomics has been successful in the isolation, description, and characterisation of new bacterial species from the human microbiota [25,27,28]. This enabled the expansion of the current human microbial database via the isolation of a significant number of novel bacterial species and allowed the identification of previously considered “uncultured organisms”, with potential for further use in clinical settings [28]. Moreover, culturomics remains as a main strategy for the isolation of new gut microorganisms with metabolising capacities. Moreover, the searching of next generation probiotics (NGP) increases due to the advancing knowledge of the human intestinal microbiota and the potential of intervening and modulating the specific dysbiosis and certain metabolic diseases [29,30].

Thus, a new area of research is worth exploring where potential NGPs with the ability to modulate the gut microbiota could be used, counteracting the impact of xenobiotics ingested through the diet. BPA measurement in foods usually consumed by children showed summatory exposure levels over 400 ng g^−1^ [10]. Moreover, animal studies detected BPA levels in faeces over 50–70 ng g^−1^ [31]. Directed culturing of microorganisms from the gut microbiota of obese and non-obese individuals exposed to EDC may lead to the identification of strains with xenobiotics detoxifying potential, which could be assessed for being used as NGP [29,32].

The aim of this work was to advance knowledge regarding culturomics data and directed culturing techniques for searching key microbiota isolates from children with obesity vs normal-weight with differential metabolic capacities. Comparative gene catalogues of the identified BPA tolerant or biodegrader taxa will be further well-defined through molecular and enzymatic pathways analyses.

## 2. Results and Discussion

### 2.1. Microbiota Culturing Approaches, Media, and Conditions for Isolation of Gut Microbial Taxa Components

Theoretical searching of culturomics data, which were thoroughly analysed, allowed us to retrieve and compile main culturing media and conditions for isolation of relevant gut microbiota taxa components (Table 1). Data analysis and extraction revealed a battery of media for successful isolation of anaerobic and aerobic species and taxa belonging to phyla Firmicutes, Bacteroidetes, Actinobacteria, and alpha-Proteobacteria. Similarly, useful information on favoured cultured isolates from gut microbiota acting as beneficial microorganisms or potential NGP was previously retrieved. Main media and pertinent modifications for isolating potential obesity and anti-obesity probiotics were: BHI, GAM, gut microbiota medium (GMM), *Lactobacillus* selection (LB), MRS, YCFA, and BPA-added media [29]. Therefore, culturomic efforts contributed to enlarge the repertoire of isolated bacterial species from humans by 28% and provided biological material to the scientific community that can be further studied for its role and interaction with other bacterial species and host [33]. Conversely, metagenomics aims to describe the human microbiota taxa without culturing efforts, given their limitations [34,35], including incomplete genomic databases [28,33,36] and the inability to distinguish between live and dead bacterial specimens in the studied samples [36]. In a previous study that examined the gut microbiota composition of eight healthy individuals, it was shown that culturomics enabled 20% higher bacterial richness in comparison to metagenomics [37]. Interestingly, the isolated species’ genome sequences were increased by 22% compared to the data obtained by metagenomic analyses alone, suggesting that the number of species recovered by culturing was higher than the number of species detected by metagenomics [37]. It is also important for culturomics approaches to highlight how faecal specimens collection, transport, and storage systems were designed and validated to optimise the viability of all groups of bacteria under several situations. After analysing the present results, the anaerobic kit used for collection and the immediate storage at −80 °C until the culturing assays, resulted in being effective in maintaining anaerobic and aerobic bacteria viability and relative abundances in all specimens analysed. Altogether, it highlights the importance of better exploring the culturing approaches to obtain microbial resources and understand the gut microbiome’s specific functional roles. In this sense, BPA-degradation capabilities of certain microorganisms were previously studied as a source for environmental bioremediation [38,39]. Furthermore, species from *Bacillus* genus isolated from infant faecal samples were shown to harbour the four complete molecular pathways for BPA biodegradation [40]. This could underline the relevance of the sporobiota (all spore-forming bacteria from microbial communities [41,42]) in the tolerance or biodegradation of xenobiotics compounds such as BPA. However, while the use of BPA-degrading microorganisms is widely extended to bioremediation, based on a previous review [30], clinical studies and trials involving beneficial microorganisms, metabolic diseases, and xenobiotic obesogens are lacking.

### 2.2. BPA Directed Culturomics Taxa Catalogue from Microbiota of Normal-Weight, Overweight, and Obese Children

A total of 192 bacterial isolates were identified from the 46 microbiota specimens in several media treated with different concentrations of BPA. Participants were categorised in children with overweight, obesity, or normal-weight according to the description made by the World Health Organisation (WHO) (Table 2).

The media BHI, MRS, RCM, GAMa, and GAMg were selected for further testing adding BPA concentrations based on higher count results after preliminary global tests with the media described in Table 1. Strains were isolated from five media supplemented with BPA: BHI (80 isolates), MRS (49 isolates), RCM (30 isolates), GAMa (18 isolates), and GAMg (15 isolates). The overall mean values estimated for colony counts were: 7 × 10^7^ CFU/g in BHI + BPA 20 ppm; 2 × 10^8^ CFU/g in BHI + BPA 50 ppm; 8 × 10^7^ CFU/g in MRS + BPA 20 ppm, and 4 × 10^7^ CFU/g in MRS + BPA 50 ppm; 5 × 10^7^ CFU/g in RCM + BPA 20 ppm, 1 × 10^7^ CFU/g RCM + BPA 50 ppm; 1 × 10^6^ CFU/g in GAMa + BPA 20 ppm, and 5 × 10^5^ CFU/g GAMa + BPA 50 ppm; 5 × 10^6^ CFU/g in GAMg + BPA 20 ppm, and 2 × 10^6^ CFU/g GAMg + BPA 50 ppm. We isolated taxa from microbiota in 25 different culture conditions (BPA directed culturing with five media and five BPA concentrations).

Similarly, current culturomics studies suggested that through using 16 culture conditions, around 98% of the known cultivated taxa of the human gut microbiota could be isolated [46]. It is interesting to highlight that Actinobacteria taxa with high BPA tolerance were isolated only from BHI medium.

The relative abundance of the isolates, together with the taxonomically closest species, the maximum BPA concentration tolerated and the specific media used for obese and normal-weight children specimens are detailed in Table 3. A phylum data analysis showed differences in relative abundance of cultured Firmicutes, Proteobacteria, and Actinobacteria between both populations. Firmicutes were the most abundant phylum with BPA tolerance found, representing 72% in normal-weight children and 73% in children with obesity. Proteobacteria was differentially represented in both groups by 17% and 20%, respectively. However, the dataset showed differences in Actinobacteria and uncultured bacterial groups. The Actinobacteria group represented 6% of the bacteria isolated from the gut microbiota of normal-weight children and 5% in the case of children with obesity. Uncultured bacteria represented 5% of the total bacteria isolated from the microbiota of normal-weight group, and 3% in population with obesity.

Similarly, xenobiotic-tolerant and specifically BPA-tolerant gut microorganisms were previously described for the traditional probiotics *Bifidobacterium breve* strain Yakult (BbY) and *Lactobacillus casei* strain Shirota (LcS), that showed protective effects against BPA dietary exposure in rats by reducing the intestinal absorption of BPA and facilitating its excretion [59]. Likewise, *Lactococcus lactis* strains adsorbed BPA but it was not able to degrade it [60]. Bioaccessibility of BPA decreased after digestion and this exposure changed the microbial community by up-regulating the abundance of BPA-degrading bacteria, such as *Microbacterium* and *Alcaligenes* [61].

The most dominant BPA-tolerant taxa found in this study were *Enterococcus* spp., *Bacillus* spp., *Escherichia* spp., *Staphylococcus* spp., and *Clostridium* spp. In both populations (Table 3), representing about 80% of the cultured microorganisms found. However, differences between the groups were observed in the minority BPA-tolerant genera cultured. Some of these genera were found exclusively to each population, demonstrating dissimilarities in gut microbiota compositions between obesity and normal-weight children. The less abundant BPA-tolerant genera found only in normal-weight children were *Rothia*, *Paraclostridium*, and *Bifidobacterium*. However, taxa belonging to *Kocuria*, *Micrococcus*, *Burholderia*, *Raoultella*, *Shigella*, and *Latilactobacillus* were found exclusively in overweight and obese children.

### 2.3. BPA Directed Culturomics and Spore-Forming Microbiota Taxa: Clostridium and Bacillus *spp.*

A total of 45 spore-forming bacterial isolates (sporobiota) from the human gut microbiota with high BPA tolerance (>20 ppm) were identified. They were isolated from five different media supplemented with different concentrations of BPA: GAMg (16 isolates), GAMa (17 isolates), and RCM (12 isolates) without any specific media for associated taxa. The overall mean values for colony counts were: 9 × 10^4^ CFU/g in GAMa + BPA 20 ppm; 4 × 10^4^ CFU/g in GAMa + BPA 50 ppm; 1 × 10^5^ CFU/g GAMg + BPA 20 ppm; 6 × 10^4^ CFU/g GAMg + BPA 50 ppm; 9 × 10^4^ CFU/g RCM + BPA 20 ppm; and 5 × 10^4^ CFU/g RCM + BPA 50 ppm. The relative abundance of these spore-forming bacterial isolates, together with taxonomically closest species, maximum BPA concentration tolerated and specific media used are detailed in Table 4. Sporobiota isolates from normal-weight children were distributed within *Clostridium* spp. Represented by 24% and *Bacillus* spp. By 27.4%. In contrast, in obese children, higher percentages were found for *Clostridium* spp. that constituted 43.9%, being similar for *Bacillus* spp. as 25.1%. It is interesting to highlight that *Paeniclostridium sordellii* showing high BPA tolerance was isolated only from normal-weight children, where it was the most representative species isolated (34.5%). Focusing on biodiversity at the species level, a total of 11 different species and 1 uncultured isolate belonged to normal-weight children. In overweight or children with obesity, a total of seven different species and three previously uncultured bacteria were successfully cultured. It is well known that traditional methods for determining the diversity of spore-forming bacteria are very important but still challenging [41]. It needs a combined approached based on culture, molecular, and omics methodologies [62,63,64].

**Figure 1:**
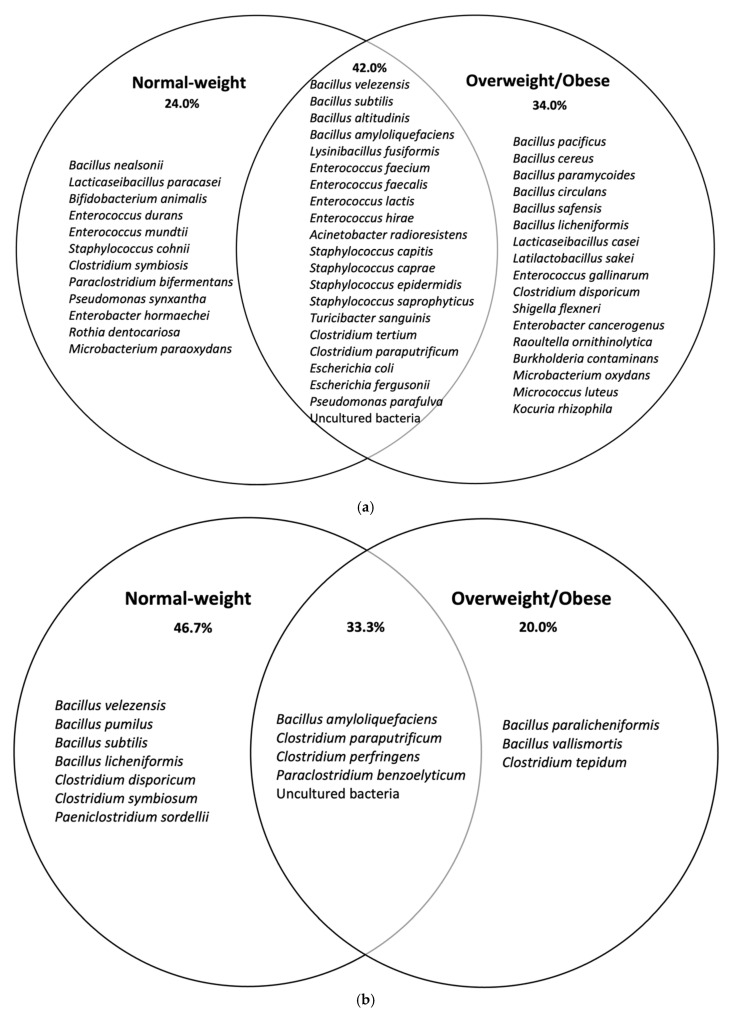
Venn diagram analyses of species isolated in normal-weight and overweight/obese children after in (**a**) BPA directed culturomics and (**b**) BPA directed culturomics spore-forming bacteria (sporobiota).

Moreover, microbial communities identified through BPA culturomics-based approach revealed that the overweight/obese group had more diversity, richness, and evenness than the normal-weight group. It was also observed that there were more differential species in obese than in normal-weight, considering global taxa analysis (Figure 1a). Conversely, sporobiota taxa analysis (Table 4) showed more variety of species in normal-weight (Figure 1b). As for other culturomic studies, these results lead to complement the molecular approaches by highlighting the role of bacteria that were considered “un-cultivable” as they might be impacting health balance and/or disease development [36]. Culturomics for isolation of new bacterial species able to metabolize toxicants was previously done by other authors, and it was framed as toxicogenomics studies [24]. New isolated bacterial species should be subjected to a series of phenotypic, biochemical, and genomic characterisation (habitat, sporulation, shape, antibiotics profile, metabolism, fatty acids contents, genome sequencing/ assembly, and annotation).

### 2.4. BPA Biodegradation Metabolic Maps through WGS^T^ Data Mining

**Figure 2:**
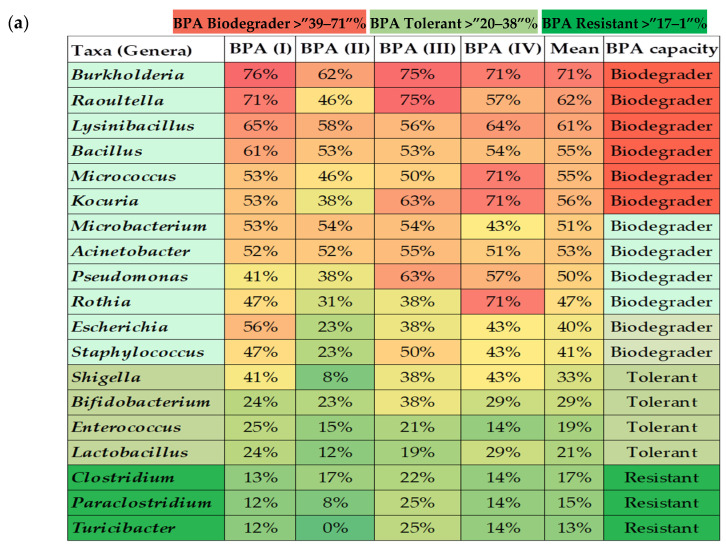

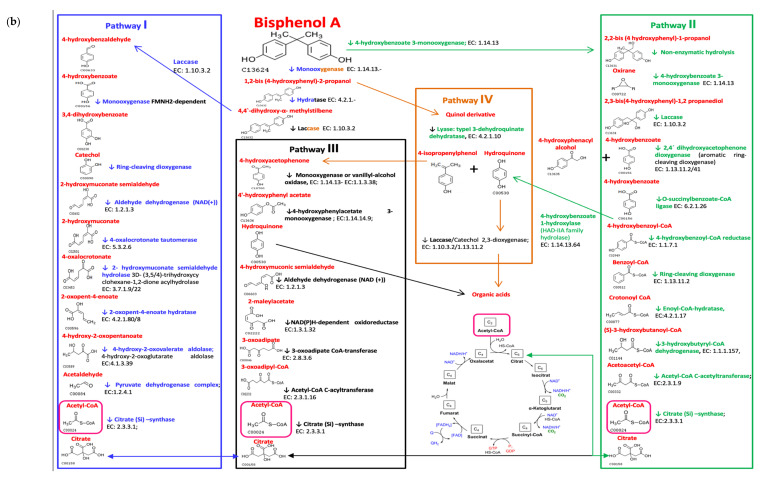
Bioinformatics and molecular predictive analysis; (**a**) percentages of BPA genes of the four different pathways present on WGS^T^ from the major isolated representative microbiota taxa, (**b**) biodegradation pathways of BPA.

**Figure 3:**
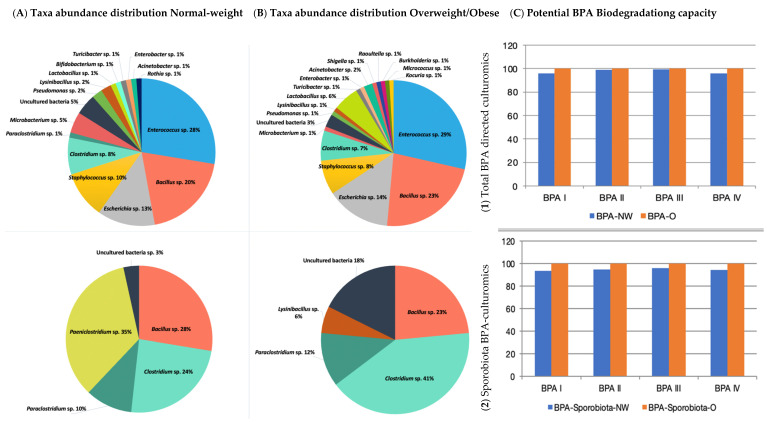
Percentages of microbial taxa abundance distribution of isolated genera after BPA treatments in Normal-weight (**A**); Overweight/Obese samples (**B**) and its Potential BPA Biodegradation capability (**C**) (proportion of BPA degradation genes) according to (**C1**) Total BPA directed culturomics (**C2**) Sporobiota BPA-culturomics approach. Normal-weight (NW); Overweight/Obese (O).

The bioinformatics and molecular analysis carried out on the WGS of type strains from the taxonomic closest species to the isolates from the gut microbiota identified as cultivable species showed a differential potential of BPA biodegradation corresponding to its specific enzyme arsenal (Figure 2). Genome mining allowed the identification of specific clusters prone to degrade bisphenols described according to the main four BPA biodegradation pathways. Bioinformatics tools and Pascal programming allowed the analysis of the retrieved sequences of the relevant type strains from a public database (GenBank—Supplementary Table S1). According to the theoretical predictive results, the overall BPA directed-cultured microbiota naturally possessed an intermediate degree of BPA biodegradation potential depending on the different enzymatic arsenal estimated (BPA (I) 47%, BPA (II) 34%, BPA (III) 48%, and BPA (IV) 50%). Specific taxa belonging to *Burkholderia*, *Bacillus*, *Raoultella*, *Acinetobacter, Micrococcus, Pseudomonas*, and *Microbacterium* could be clustered as biodegrader because they harboured the more complete BPA biodegradation genetic clusters (>50%). Conversely, *Clostridium* species could be considered as BPA resistant bacteria. Similarly, *Shigella*, *Bifidobacterium*, *Enterococcus*, *Lactobacillus,* and *Turicibacter* were considered tolerant or resistant as the predictive analysis showed lower representative percentages of the gene loci for encoding BPA biodegradation enzymes. After crossing data from the relative abundance data from cultured of isolated taxa with their specific BPA biodegradation predictive values, a lower score was found in normal-weight compared to overweight or obese populations (Figure 3a). The same trend was also observed when analysing the sporobiota alone and its BPA genes potential (Figure 3b). Remarkably, the analyses results showed that the composition of independent sporobiota taxa (mainly *Bacillus* spp.) might contribute to a significant role in the gut dysbiosis/eubiosis on the studied children population. It seems very important to elucidate the differential impact of specific metabolites from the BPA pathways that can specifically contribute to trigger obesogenic effects, such as Acetyl CoA, which is a convergent end metabolite in several BPA pathways, and is demonstrated to have obesogenic effects [65,66].

Genome mining of WGS^T^ for BPA biodegradation genes was performed through available databases. Advances in Next Generation Sequencing (NGS) and in silico tools allowed us to perform an appropriate screening for genes of concern in the gut microbiota. BPA biodegradation capacity or toxicomicrobiomics more generally can potentially be exploited through bioinformatics, metagenomics, or in silico analysis of cultivable isolates via WGS [67,68]. Moreover, a better understanding of the microbiota ecology driven by metabolites and bioactive compounds, which are released by specific gut microbial components, may drive towards better and personalised clinical interventions [69]. Genome mining conducted in the present study allowed BLAST driven searching for predicted BPA pathways. Pascal programming was a useful prediction tool for toxicomicrobiomics analysis. Similarly, another useful prediction tool could be used as well as for BPA biodegradation pathways [70].

Interestingly, certain species found exclusively in the microbiota of normal-weight children microbiota (*Paraclostridium* spp. and *Bifidobacterium* spp.) had a low BPA biodegradation potential, being clustered as BPA tolerant or resistant. However, certain species isolated exclusively from the microbiota of overweight and/or obese children (*Kouria* spp., *Micrococcus* spp., *Burkholderia* spp., *Raoultella* spp., and *Shigella* spp.) showed the higher BPA degradation potential, being grouped as BPA biodegrader. Thus, a first trend of this analysis revealed that microorganisms from the gut microbiota of children with obesity may have more BPA biodegradation potential than those of normal-weight children.

Based on comparative data from metagenomics regarding the variability of taxa composition in individuals with obesity and normal-weight, the Firmicutes/Bacteroidetes (F/B) ratio constitutes a recognised biomarker for comparisons, as well as the relative abundances of Actinobacteria and Proteobacteria. In the present study, four major bacterial phyla (Firmicutes, Bacteroidetes, Proteobacteria, and Actinobacteria) dominated the gut microbiota and a predisposition of higher relative abundance of Firmicutes and Actinobacteria in the individuals with obesity was observed. The landscape, richness, and diversity of the observed study group showed no statistical significance in the variability of taxa composition between individuals with obesity and normal-weight. For the statistical analysis of comparative metagenomics of the cohort, the microbiome data were uploaded to the MicrobiomeAnalyst server [71]. Since microbiome studies usually generate datasets that are both large in size and complex in structure, and could carry a great ‘big data’ challenge in downstream data analysis, the platforms such as the MicrobiomeAnalyst server are powerful tools, which offer comprehensive support with a wide array of methods for taxonomic diversity analysis, functional profiling, visualisation, and significance testing. The F/B ratio was higher in obese than normal-weight individuals, and Actinobacteria were usually more abundant in an obese population. Conversely, Bacteroides and Proteobacteria were slightly higher in normal-weight populations [72]. In this sense, our BPA directed culturomics approach highlighted Firmicutes as one of the most predominant populated taxa able to grow with high BPA concentrations and relatively high BPA biodegradation potential (*Bacillus*, *Staphylococcus*, *Micrococcus*). However, it was not possible to explore Bacteroidetes in a similar way, as no cultivable components of this taxon were obtained through this approach. On the other hand, cultivable Proteobacteria taxa (Table 2) isolated from obese specimens were different compared to those obtained from normal-weight children. Moreover, they harboured the highest BPA enzymatic capacities according to the gene loci presence (*Burkholderia contaminans, Raoultella ornithinolytica, Acinetobacter radioresistens Escherichia* spp., and *Shigella flexneri*).

Moreover, it is important to consider the ecological role of these enzymes and their impact on the composition of the gut microbiota, which may have a large influence on metabolising and neutralising BPA via releasing metabolites that contribute to the modification of individual microbial taxa components on a long-term basis [73]. Interestingly, specific transitory taxa of the gut microbiota with a high potential of BPA biodegradation could also be used for environmental bioremediation purposes or as animal or plant probiotics. Similarly, several authors investigated the BPA removal capacity of bacterial strains isolated in dessert soil, including *Pseudomonas putida*, *Pseudomonas aeruginosa*, *Enterobacter cloacae*, *Klebsiella* sp., and *Pantoea* sp. [8,74]. Likewise, a consortium of microorganisms isolated from river sediment (*Terrimonas pekingensis* and *Pseudomonas* sp.) was demonstrated to use BPS as the sole carbon source and was highly efficient in degrading 99% of the BPS with an initial concentration of 50 mg/L in 10 days [75]. Moreover, gut bacteria harbouring laccases could be used for detoxification of several hazardous dietary contaminants and emerging EDC in a bioreactor with a novel biocatalytic system based on active membranes and immobilised laccase technology [76]. The directed culturing approach could be also applied to study and predict the microorganisms able to biodegrade other obesogens and MDCs, such as parabens, phthalates, and benzophenones [77,78,79].

## 3. Materials and Methods

### 3.1. Culturomics Review Data for Increasing the Microbiota Taxa Isolates

A literature search and review of studies was conducted in collaboration with Granada library support, using medical subject headings (MeSH) and key words (see below) under a stepwise procedure search adapted to each database’s tutorials. The search was limited for culturomics literature published until July 2021, and conducted on the following electronic databases: PubMed, Web of Science (Thomson Reuters Scientific), and Scopus (Elsevier). Titles and abstracts were reviewed, then full-text publications with reference to the inclusion criteria, which were all the studies about culturomics or culturing from human gut microbiota. The key words were culturomics* AND microbiota, culturing* AND microbiota AND obesity AND “endocrine disrupt*”; culturomics* and microbiota and obesity and xenobiotic*; culturing * and microbiota and obesity and hormon*; culturing* and microbiota and obesity and “drug metabol*”; culturing* and microbiota and “metabolic syndrome” “endocrine disrupt*”; culturomics* and microbiota and “metabolic syndrome” and xenobiotic*; culturomics* and microbiota and “metabolic syndrome” and hormon*; culturomics * and microbiota and “metabolic syndrome” and “drug metabol*”; culturomics * and microbiota and diabetes and “endocrine disrupt*”; culturomics * and microbiota and diabetes and xenobiotic*; culturomics* and microbiota and diabetes and hormon*; culturomics* and microbiota and diabetes and “drug metabol*”; culturomics* and microbiota and fertility.

### 3.2. Experimental Culturomics Approach to Isolate Gut Microbes Metabolising Obesogenic EDCs

#### BPA Directed Culturing Approach for the Isolation of Microbiota Strain Catalogue

A previous common approach to isolate microbial strains from microbiota after BPA treatment has been followed [40]. For this study, human faecal samples were collected with anaerobic kits from obese, overweight, and normal-weight children aged between four and twelve years old, anthropometry data are detailed in Table 2. The anthropometry classification was made according to data collected by WHO 2007 (Table S2, https://www.who.int/toolkits/growth-reference-data-for-5to19-years/indicators/bmi-for-age 22 October 2021). The faecal samples were collected in anaerobic conditions and maintained appropriately frozen at −80 °C until experimental assays were performed for avoiding significant loss of isolated taxa as previously was described in several studies [80,81].

Faecal samples (0.5 g) underwent a directed culturing approach adding BPA to search tolerant and potentially BPA biodegrading microorganisms. The experiment was carried out by serial dilution method of the samples and their exposition to different BPA concentrations (0.5, 10, 20, and 50 ppm) during 72 h at 37 °C, accordingly to previous primary searching and screening studies to obtain microbial biodegrader strains [40]. Further spreading in different media was performed and later incubated under aerobic and anaerobic conditions with anaerobic jars through Anaerocult^®^ A system (Merck, Darmstadt, Germany) at 72 h and 37 °C. The different culture media used for optimising the uncultured bacterial growth were brain heart infusion (BHI), Man, Rogosa and Sharpe (MRS), reinforced clostridial medium (RCM), Gifu anaerobic modified medium (GAMm) agar/gellan [82]. The isolation of BPA-tolerant bacteria was carried out under the experienced picking method [43], according to different morphology of the colonies, these were isolated as pure culture for subsequent morphological, phenotypic, and genotypic identifications.

### 3.3. Genomic DNA Extraction and Partial and Complete 16S rRNA Analysis

Microbial genomic DNA was extracted using DNeasy columns following the manufacturing instructions, and DNA extraction from stools was performed using the PowerSoil DNA Isolation Kit (Qiagen^®^, Hilden, Germany). The isolated DNA was quantified using Nanodrop (Thermo Scientific, Waltham, MA, USA) and biophotometer (Eppendorf^®^ D30). The quality of DNA was analysed spectrophotometrically, by gel electrophoresis. Partial and complete 16S rRNA genes from gDNA from all isolated colonies were amplified by PCR and subsequently sequenced by Sanger method and analysed to identify each taxon (primer amplification: 16F27-5′-AGAGTTTGATCMTGGCTC-3′ and 1525R-5′-AAGGAGGTGATCCAGCC-3′; primers sequencing: F357-5′-CTCCTACGGGAGGCAGCA-3, R519-5′-GWATTACCGCGGCKGCTG-3, and F915-5′-GGGCCCGCACAAGCGGTGG-3) (Institute of Parasitology and Biomedicine “López-Neyra” (IPBLN) Service). Sequence analysis was done using Chromas Pro 2.0 (Technelysium Pty Ltd., Tewantin, Australia). Sequences were examined for maximum homology against GenBank using the National Centre of Biotechnology Information’s (NCBI) BLASTn program. The collection and phylogenetic comparison of 16S rRNA partial gene sequences was done using the Ezbiocloud platform [83].

### 3.4. BPA Directed Culturing and Searching for Spore-Forming Taxa Components: Clostridium *spp.* and Bacillus *spp.*

In parallel, a specific treatment was carried out to favour the isolation of spore-forming bacteria. For this, after the exposure to BPA and before the spread on the media, the microbiota samples were exposed to 70% ethanol for 4 h and treated with a bile acids solution (0.1 mg/mL of bile bovine in PBS) for the metabolic activation of the spores. Then, the samples were processed and analysed as described above.

### 3.5. Genome Data Mining Tools for Prediction of BPA Metabolic Maps and Enzymatic Pathways in Whole Genome Sequence of Type Strains (WGST) from the Closest Species to Isolates from Gut Microbiota

In order to assess the presence of BPA biodegradation gene potential of the cultured microbiota, several bioinformatics tools were used to perform the genome mining.

The identification of potential BPA genes encoding enzymes involved in the four biodegradation pathways was carried out by the analysis of genomes of type strains WGS^T^ closest to the representative isolates identified. A data retrieving program was specifically computed using Pascal programming language, to obtain the identifier ID of the specific enzymes participating in the BPA pathways and the corresponding loci from the microbial genomes (Figure 2).

The type strains whole genome sequences (WGS^T^) were retrieved from NCBI Genome Data Bank as GenBank file format to list the genes that could potentially encode the enzymes of interest. A more detailed prediction of the BPA gene clusters from the four main pathways was performed by checking the downstream and upstream genes of those involved in BPA biodegradation, using NCBI genome map viewer.

Pascal programming language registers the entire GenBank file of the type species and checks whether a certain keyword for the enzyme was found in the field corresponding to the name of the protein. The program returns in a notepad file a list of the possible relevant candidate enzyme names and identifier ID, loci, and annotations features. Afterwards, the output file notes are reviewed, validated, and organized through checking the presence or absence of the relevant enzymes, either of any of the degradation routes of BPA whose presence was searched. An Excel spreadsheet shows a positive (+) if the microorganism is theoretically capable of producing that enzyme, or a negative (−) if was not able to produce it (Supplementary Table S1). Verification of certain enzyme names according to their function and substrate was also done using different nomenclatures supported by the Kyoto Encyclopaedia of Genes and Genomes database (KEGG Enzyme, https://www.genome.jp/kegg/annotation/enzyme.html 24 June 2021).

## 4. Conclusions

The pathophysiological impact and severity of obesogens, such as BPA, appears to depend on inter-individual and diverse gut microbial components that trigger microbiota dysbiosis with clinical symptoms of obesity and metabolic disorders. Therefore, to contribute to the understanding of how these specific microbial consortia interact with the host and how their enzymatic arsenal could shape those communities and functional human microbiome become a relevant health challenge. Innovatively, BPA directed culturing and molecular approaches indicated that specific gut microbiota found in normal-weight vs obese individuals were differentially enriched in potential biodegrader, resistant, and tolerant microorganisms. The present study also highlights the potential biodegradation of the sporobiota, focused mainly on *Bacillus* genus as relevant for impacting on the obesogenic phenotypes. Moreover, directed-cultured microbial communities from children with obesity showed a slight higher potential capacity of BPA genes encoding enzymes compared to the normal-weight population. It should be further investigated how the differential microbiota components release variable BPA metabolites or intermediates that might play determinant roles on obesity. In addition, cumulative environmental exposure to these obesogens might also drive microbiota ecological modifications that trigger dysbiosis and differential expression of specific gene pathways, impacting on the long-term individual health and disease status.

## Figures and Tables

**Table 1:** Culturing media and conditions for isolation of microbiota taxa components (Aer: aerobic; AAn: aerotolerant anaerobe; SAn: strictly anaerobic; FAn: facultative anaerobe).

Species/Oxygen Tolerance		Culturing Media and Conditions *
Firmicutes	*Bacillus* spp./Aer/AAn /Fan [25,43,44,45,46]	BCB38; BCB02; BCB03; BCB04; BCB05; BCB06; BCB08; BCB09; BCB10; COS01; COS03; MB01; MB02; TSB01; BHI01; BCB19; YCFA06; BCB23; COS09; TSB04; BCB07; YCFA02; MB03; TSB03; BCB18; BCB01; COS02; COS04; BHI02; CBA01; MRS02; BCB37; BCB33; BHI07; BCB36; BCB46; BCB14; BCB15; BCB12; BBCB22; BHI04; BCB13; YCFA01; MB04; BCB55
	*Blautia* spp./SAn [44,45,46,47,48,49,50]	BHI05; BCB13; BCB15; BCB01; BCB03; BCB05; BCB07; BCB09; BCB10; COS02; MB02; TSB04; YCFA05; CBA01; BCB52; RM01; BCB11; CNA01; YCFA01; BCB28; BCB19; COS09; YCFA03; WC02; CBA02; GAM02; RCA02
	*Clostridium* spp./San [27,43,46,47,51,52,53]	BCB01; BCB03; BCB05; BCB07; BCB09; BCB10; COS04; MB02; BCB15; COS02; RM01; RCA01; BCB34; BCB39; CHRIS01; CBA01; BCB19; COS09; YCFA05; BCB13; TSB04; CBA02; YCFA01; SCM04; YCFA04; MB04; RM02; BCB49; BCB28; BCB25; CNA01; BCB17; BCB21; BCB50; BCB32; BCB02; BCB04; BCB11; WC01; BHI02; YCFA03; RCA02; WC02; BHI03; BCB33; BCB30; TSB02; BCB31; YCFA02; MRS02; RM03; COS03; TSB01; BCB22; COS08; MB03; TSB03; BCB06; MRS01; BHI01; BCB23; BCB12; BCB14; BCB16; BCB20
	*Dialister* spp./SAn [46]	BCB07; CHRIS01; SCM04; RM01; RM02; BCB11; BCB19; COS02; BCB01; BCB03; BCB05; BCB09; BCB10; COS04; MB02; YCFA01; MRS01
	*Enterococcus* spp./FAn [43,46,54]	CBA03; YCFA04; YCFA06; BCB04; BCB07; BCB06; BCB08; COS01; COS03; COS04; MB01; MB02; TSB01; YCFA01; CHRIS01; MRS01; SCM04; RM01; BCB23; BCB11; BCB17; BCB22; BCB19; BCB20; COS09; MB03; TSB03; TSB04; BCB10; RM02; BCB01; BCB03; COS02; BCB05; BCB09; BHI01; BCB02; CNA01; RM03; SCM01; YCFA02; RCA01; BCB15; BCB21; TSB02; WC01; BHI02; CBA01; MRS02; BCB13; BCB14; COS08; MB04; BCB12; BCB16; YCFA03; RCA02; WC02; BHI03; MRS03
	*Eubacterium* spp./SAn [43,46]	BCB07; SCM04; BCB15; MB02; BCB19; BCB01; BCB05; BCB09; COS02; COS04; RM01; RCA01; YCFA04; BCB03; BCB11; WC01; CBA01; COS09; BCB13; MB04
	*Lactobacillus* spp./AAn [46]	BCB07; COS04; SCM04; CNA01; BCB10; COS02; YCFA01; MRS01; RM01; BCB11; YCFA02; CHRIS01; BCB15; BCB19; COS09; BHI03; BCB02; BCB03; BCB04; BCB06; COS01; COS03; MB01; MB02; TSB01; BHI01; BCB13; RCA01; RM02; BCB23; RM03; SCM01; CBA01; MRS02; BCB01; BCB09; BCB05; WC01; BHI02
	*Megasphaera* spp./SAn [46,55]	COS02; COS04; RM01; BCB07; YCFA01; BCB09; BCB10; SCM04; BCB31
	*Peptoniphilus* spp./San [25,27,46]	CHRIS01; BCB01; BCB05; BCB07; MB02; BCB10; COS02; COS04; RM02; BCB35; YCFA01; MRS01; RM01; BCB53; BCB15; BCB03; BCB09; SCM04; BCB11; BCB38; BCB40; YCFA03
	*Ruminococcus* spp./SAn [25,27,46,47]	YCFA05; BCB11; RM03; SCM01/04; YCFA02; CBA01; BCB05/11/13/15/40/41; BCB19; BCB03; BCB07; BCB09; COS02; RCA02; BHI03; RM01; RM02; TSB04; YCFA01; CHRIS01; CNA01
	*Staphylococcus* spp./FAn [43,46]	YCFA06; BCB01; BCB02; BCB03; BCB07; BCB06, BCB10; COS01; COS04; MB01; MB02; YCFA01; RM01; RM02; BCB11; BCB15; BCB19; COS09; MB04; BCB05; BCB14; BCB17; BCB20; COS08; MB03; TSB03; BCB08; BCB09; CHRIS01; BCB04; COS02; COS03; BHI01; CBA01
	*Streptococcus* spp./FAn [43,46]	BCB07; YCFA04; BCB04; BCB05; BCB10; MB02; RM01; CBA01; BCB06; COS02; BHI01: YCFA01; BCB02; BCB09; COS01; COS03; BCB03; COS04; BCB23; CNA01; BCB01; CHRIS01; SCM04; BHI02; BCB08; TSB01; MRS01; RCA01; WC01; YCFA06; BCB11
Bacteroidetes	*Alistipes* spp./SAn [25,27,43,46,47]	YCFA05; BCB01; BCB03; BCB05; BCB07; BCB09; BCB10; COS02; COS04; RM02; BCB11; CNA01; YCFA02; WC01; CBA01; BCB19; YCFA01; CHRIS01; SCM04; BCB48; MB02; BHI02; CPVX01; BCB13; BCB24; MRS01; TSB04; YCFA04; RCA01; BRU02; SCM01; BCB15; COS09; MB04; SCM02; RM03; BCB27
	*Bacteroides* spp./SAn [27,43,46]	BCB01; BCB03; BCB05; BCB07; BCB09; BCB10; COS02; COS04; MB02; BCB11; SCM01; YCFA02; RCA01; WC01; BHI02; CBA01; MRS02; BCB19; RM03; BCB13; CBA02; SCM04; YCFA04; CNA01; RM01; TSB04; BCB15; RM02; YCFA01; CHRIS01; MB04; TSB03; WC02; COS09; YCFA03; TSB02
	*Butyricimonas* spp./SAn [27,43,46]	BCB41; BCB01; BCB03; BCB05; BCB07; BCB09; BCB10; COS02; MB02; CBA01; YCFA04; CHRIS01; SCM04; RM02; BCB11; SCM01; COS09; YCFA02; CNA01; BCB19
	*Parabacteroides* spp. /SAn/[43,46]	BCB05; BCB07; COS02; COS04; SCM04; RM02; CBA01; BCB19; YCFA04; CHRIS01; RM01; BCB11; CNA01; SCM01; BCB15; TSB04; BCB01; BCB03; BCB09; BCB10; BHI01; WC01; BHI02; YCFA01; MB02; YCFA02; RCA01
	*Prevotella* spp./SAn [43,46,47,56]	BCB10; COS02; RM01; BCB05; BCB01; BCB07; BCB09; YCFA01; CHRIS01; WC01; BCB11; CNA01; CBA01; YCFA05; SCM01; SCM04; CBA04; BRU03; BCB19; BCB03
Actinobacteria	*Actinomyces* spp. AAn [25,27,47]	BCB03; BCB09; YCFA02; CBA01; COS09; BCB19; BCB07; MB02; BCB48; BCB11; BCB42
	*Bifidobacterium* spp. /An/SAn [43,46]	BCB07; BCB10; YCFA01; MRS01; SCM04; RM01; RM02; BCB11; CNA01; RM03; SCM01; CBA01; BCB15; BCB19; COS09; BCB01; BCB03; BCB05; YCFA02; RCA01; WC01; BHI02; COS02; MB02; CHRIS01; MRS02; BCB13; BCB17; YCFA03; WC02; BHI03; CBA02; MRS03; RCA02; BCB09; COS04; YCFA04; BCB23
	*Collinsella* spp./SAn [27,43,46]	YCFA04; BCB05; BCB07; COS02; YCFA01; CHRIS01; RM01; BCB11; CNA01; RM03; SCM01; CBA01; BCB13; BCB15; BCB19; SCM04; RM02; BCB01; CBA02; MRS02; BCB41; MB07; BCB10; YCFA02; BCB23; COS09
	*Corynebacterium* spp./AAn [27,46,57]	SCM04; RM02; BCB11; BCB23; CPVX02; COS02; BCB44; BHI01; BCB07; COS04; BCB10; MRS01; CBA03
	*Propionibacterium* spp. /AAn [46]	BCB07; YCFA01; RM02; BCB11; BCB19; MB04; TSB04; YCFA03; CHRIS01; MRS01; SCM04; RM01; BCB09; MRS02; BCB02; BCB06; BCB10; MB01; MB02
α-P	*Enterobacter* spp./AAn [43,46,58]	BHI08; COS04; MB02; RM01; RM02; YCFA04; MRS01; BCB23; YCFA06; BCB11; BCB02; BCB03; BCB04; BCB07; BCB09; COS01; COS02; MB01; BHI01

* BCB: blood culture bottle; BHI: brain heart infusion; BRU: Brucella medium; CBA: Columbia blood agar; CHRIS: Christensenella medium; COS: Columbia agar liquid medium + 5% sheep blood; CPVX: chocolate agar + PolyViteX; GAM: Gifu anaerobic media; GAMa: Gifu anaerobic media agar; GAMg: Gifu anaerobic media gellan; MB: marine broth; MRS: Man, Rogosa, and Sharpe; RM: R-medium; RCA: reinforced clostridial agar; RCM: reinforced clostridial media; SCM: Schaedler medium; TSB: trypticase soy broth; YCFA: yeast extract-casein hydrolysate-fatty acids; WC: Wilkins Chalgren.

**Table 2:** Anthropometry global data from children with obesity and normal-weight individuals categorised by WHO.

Normal-Weight Children *						Overweight/Obese Children *					
Female			Male			Female			Male		
ID	BMI	Age	ID	BMI	Age	ID	BMI	Age	ID	BMI	Age
1	17.1	7	13	13.8	6	23	19.6	7	35	22.3	9
2	18.4	12	14	13.2	5	24	20.7	10	36	22.1	8
3	15.6	6	15	15.9	5	25	21.2	8	37	25.4	10
4	15.6	6	16	15.3	7	26	23.9	8	38	17.4	6
5	13.9	8	17	15.6	7	27	18.2	8	39	20.6	6
6	13.8	6	18	14.1	5	28	22.6	12	40	23.6	11
7	17.1	6	19	14.7	4	29	18.4	11	41	24.9	6
8	14.1	4	20	14.5	7	30	25.3	10	42	23.9	11
9	15.4	6	21	18.0	9	31	26.8	10	43	21.4	5
10	14.4	10	22	16.5	10	32	29.9	7	44	27.1	8
11	13.4	6				33	26.9	11	45	25.6	9
12	16.3	6				34	21.4	9	46	23.5	9
Means ± SD 15.4 ± 1.6		6.9 ± 2.2		15.2 ± 1.4	6.5 ± 1.9		22.9 ± 3.7	9.3 ± 1.7		23.1 ± 2.6	8.2 ± 2.0

* According to WHO 2007 (data available as Table S2). ID sample, BMI (kg/m^2^), age (years), mean ± SD.

**Table 3:** BPA-tolerant cultured bacteria taxa from the gut microbiota of normal-weight and overweight/obese.

Closest Taxa	Microbiota Isolates from Normal-Weight			Microbiota Isolates from Overweight/Obese		
Phylum/Species	BPA [ppm]	Media	%	BPA [ppm]	Media	%
**Firmicutes**						
*Bacillus amyloliquefaciens*	100	BHI	1.1	20	RCM	1.0
*Bacillus velezensis*	50	BHI/MRS	11.5	50	BHI/MRS	12.4
*Bacillus subtilis*	50	BHI/MRS	3.4	50	GAMg	1.9
*Bacillus nealsonii*	50	GAMg	1.1	-	-	-
*Bacillus altitudinis*	50	BHI	2.3	20	GAMg	1.0
*Bacillus pacificus*	-	-	-	50	GAMa	1.0
*Bacillus cereus*	-	-	-	20	MRS	1.0
*Bacillus paramycoides*	-	-	-	20	RCM	1.0
*Bacillus circulans*	-	-	-	50	MRS	1.0
*Bacillus safensis*	-	-	-	20	BHI	1.9
*Bacillus licheniformis*	-	-	-	20	MRS	1.0
*Lacticaseibacillus paracasei*	10	RCM	1.1	-	-	-
*Lacticaseibacillus casei*	-	-	-	20	MRS	1.9
*Latilactobacillus sakei*	-	-	-	10	MRS	3.8
*Lysinibacillus fusiformis*	50	MRS	2.3 (1)	50	BHI	1.0
*Bifidobacterium animalis*	20	MRS	1.1 (19)	-	-	-
*Enterococcus faecium*	50	BHI/MRS/GAMg/RCM	14.9	20/50	BHI/MRS/GAMa	18.1
*Enterococcus faecalis*	20	BHI	5.7	20	BHI/RCM	2.9
*Enterococcus durans*	50	GAMg	1.1	-	-	-
*Enterococcus mundtii*	20	GAMg	1.1	-	-	-
*Enterococcus lactis*	20	RCM	1.1	50	BHI	3.8
*Enterococcus hirae*	50	RCM	3.4	20	BHI	2.9
*Enterococcus galinarum*	-	-	-	50	BHI	1.0
*Staphylococcus capitis*	20	BHI	1.1	10	MRS	1.0
*Staphylococcus caprae*	50	MRS	2.3	50	BHI	1.0
*Staphylococcus cohnii*	50	BHI/GAMa	2.3	-	-	-
*Staphylococcus epidermidis*	50	RCM	3.4	20/50	BHI/RCM/GAMa	4.8
*Staphylococcus saprophyticus*	50	BHI	1.1	20	BHI	1.0
*Turicibacter sanguinis*	50	BHI	1.1	50	BHI	1.0
*Clostridium tertium*	20	BHI	1.1	50	BHI	1.0
*Clostridium symbiosis*	20	BHI	1.1	-	-	-
*Clostridium paraputrificum*	50	BHI/GAMa	5.7	50	GAMg	4.8
*Clostridium disporicum*	-	-	-	20	GAMg	1.0
*Paraclostridium bifermentans*	50	RCM	1.1	-	-	-
**Proteobacteria**						
*Escherichia coli*	20/50	BHI/MRS/GAMa	11.5	20/50	BHI/MRS	13.3
*Escherichia fergusonii*	20	BHI	1.1	10	BHI	1.0
*Shigella flexneri*	-	-	-	50	RCM	1.0
*Pseudomonas synxantha*	20	GAMg	1.1	-	-	-
*Pseudomonas parafulva*	50	GAMa	1.1	50	MRS	1.0
*Enterobacter hormaechei*	50	RCM	1.1	-	-	-
*Enterobacter cancerogenus*	-	-	-	20	RCM	1.0
*Acinetobacter radioresistens*	20	RCM	1.1	10	BHI	1.9
*Raoultella ornithinolytica*	-	-	-	50	BHI	1.0
*Burkholderia contaminans*	-	-	-	20	MRS	1.0
**Actinobacteria**						
*Rothia dentocariosa*	100	BHI	1.1	-	-	-
*Microbacterium paraoxydans*	20/50	BHI	4.6	-	-	-
*Microbacterium oxydans*	-	-	-	50	BHI	1.0
*Micrococcus luteus*	-	-	-	20	BHI	1.0
*Kocuria rhizophila*	-	-	-	20	BHI	1.0
Uncultured bacteria	20/100	RCM/BHI	4.6	20/50	BHI	2.9

**Table 4:** BPA tolerant spore-forming bacteria taxa cultured from normal-weight and overweight/obese microbiota.

Closest Taxa	Microbiota Isolates from Normal-Weight			Microbiota Isolates from Overweight/Obese		
Sporobiota Species	BPA [ppm]	Media	%	BPA [ppm]	Media	%
*Bacillus amyloliquefaciens*	20/50	GAMa/GAMg	10.3	50	RCM/GAMa	12.5
*Bacillus vallismortis*	-	-	-	50	RCM	6.3
*Bacillus velezensis*	20/50	GAMa	6.9	-	-	-
*Bacillus pumilus*	50	GAMa	3.4	-	-	-
*Bacillus subtilis*	50	GAMa	3.4	-	-	-
*Bacillus licheniformis*	20	RCM	3.4	-	-	-
*Bacillus paralicheniformis*	-	-	-	50	GAMg	6.3
*Clostridium disporicum*	50	GAMg	3.4	-	-	-
*Clostridium paraputrificum*	20/50	RCM/GAMa/GAMg	13.8	20/50	GAMa/GAMg	31.3
*Clostridium perfringens*	50	GAMa	3.4	20	GAMg	6.3
*Clostridium symbiosum*	20	RCM	3.4	-	-	-
*Clostridium tepidum*	-	-	-	20	GAMa	6.3
*Paraclostridium benzoelyticum*	50	GAMa/GAMg	10.3	20/50	GAMg	12.5
*Paeniclostridium sordellii*	20/50	RCM/GAMa/GAMg	34.5	-	-	-
Uncultured bacteria	20	GAMa	3.4	50	GAMa/GAMg/RCM	18.9

Pre-treatment ethanol and bile acids. All sequences were submitted to GenBank under accession numbers: MZ612806-MZ612850.

## Supplementary Materials

The following supporting information can be downloaded at: https://www.mdpi.com/article/10.3390/nu14020241/s1, Supplementary Table S1: Bioinformatics for BPA enzyme ID and Loci Prediction. Supplementary Table S2: Categorisation of data for overweight, obesity, or normal-weight in children according to the description by the World Health Organisation (WHO).
