# Supplementary material for: Culturing and Molecular Approaches for Identifying Microbiota Taxa Impacting Children’s Obesogenic Phenotypes Related to Xenobiotic Dietary Exposure
Source: Nutrients. 2022 Jan 6;14(2):241. doi: 10.3390/nu14020241 (PMC8778816; doi:10.3390/nu14020241)
Supplement: Supplementary file 1 [file nutrients-14-00241-s001.zip › supple/Supplementary Table S2 Categorisation of data for overweight, obesity, or normal-weight in children according to the description by the World Health Organisation (WHO).pdf]

Simplified field tables

BMI-for-age GIRLS
5 to 19 years (z-scores)

World Health Organization

Year: Month	Months	-3 SD	-2 SD	-1 SD	Median	1 SD	2 SD	3 SD
5: 1	61	11.8	12.7	13.9	15.2	16.9	18.9	21.3
5: 2	62	11.8	12.7	13.9	15.2	16.9	18.9	21.4
5: 3	63	11.8	12.7	13.9	15.2	16.9	18.9	21.5
5: 4	64	11.8	12.7	13.9	15.2	16.9	18.9	21.5
5: 5	65	11.7	12.7	13.9	15.2	16.9	19.0	21.6
5: 6	66	11.7	12.7	13.9	15.2	16.9	19.0	21.7
5: 7	67	11.7	12.7	13.9	15.2	16.9	19.0	21.7
5: 8	68	11.7	12.7	13.9	15.3	17.0	19.1	21.8
5: 9	69	11.7	12.7	13.9	15.3	17.0	19.1	21.9
5: 10	70	11.7	12.7	13.9	15.3	17.0	19.1	22.0
5: 11	71	11.7	12.7	13.9	15.3	17.0	19.2	22.1
6: 0	72	11.7	12.7	13.9	15.3	17.0	19.2	22.1
6: 1	73	11.7	12.7	13.9	15.3	17.0	19.3	22.2
6: 2	74	11.7	12.7	13.9	15.3	17.0	19.3	22.3
6: 3	75	11.7	12.7	13.9	15.3	17.1	19.3	22.4
6: 4	76	11.7	12.7	13.9	15.3	17.1	19.4	22.5
6: 5	77	11.7	12.7	13.9	15.3	17.1	19.4	22.6
6: 6	78	11.7	12.7	13.9	15.3	17.1	19.5	22.7
6: 7	79	11.7	12.7	13.9	15.3	17.2	19.5	22.8
6: 8	80	11.7	12.7	13.9	15.3	17.2	19.6	22.9
6: 9	81	11.7	12.7	13.9	15.4	17.2	19.6	23.0
6: 10	82	11.7	12.7	13.9	15.4	17.2	19.7	23.1
6: 11	83	11.7	12.7	13.9	15.4	17.3	19.7	23.2
7: 0	84	11.8	12.7	13.9	15.4	17.3	19.8	23.3
7: 1	85	11.8	12.7	13.9	15.4	17.3	19.8	23.4
7: 2	86	11.8	12.8	14.0	15.4	17.4	19.9	23.5
7: 3	87	11.8	12.8	14.0	15.5	17.4	20.0	23.6
7: 4	88	11.8	12.8	14.0	15.5	17.4	20.0	23.7
7: 5	89	11.8	12.8	14.0	15.5	17.5	20.1	23.9
7: 6	90	11.8	12.8	14.0	15.5	17.5	20.1	24.0

BMI-for-age GIRLS
5 to 19 years (z-scores)

World Health Organization

Year: Month	Months	-3 SD	-2 SD	-1 SD	Median	1 SD	2 SD	3 SD
7: 7	91	11.8	12.8	14.0	15.5	17.5	20.2	24.1
7: 8	92	11.8	12.8	14.0	15.6	17.6	20.3	24.2
7: 9	93	11.8	12.8	14.1	15.6	17.6	20.3	24.4
7: 10	94	11.9	12.9	14.1	15.6	17.6	20.4	24.5
7: 11	95	11.9	12.9	14.1	15.7	17.7	20.5	24.6
8: 0	96	11.9	12.9	14.1	15.7	17.7	20.6	24.8
8: 1	97	11.9	12.9	14.1	15.7	17.8	20.6	24.9
8: 2	98	11.9	12.9	14.2	15.7	17.8	20.7	25.1
8: 3	99	11.9	12.9	14.2	15.8	17.9	20.8	25.2
8: 4	100	11.9	13.0	14.2	15.8	17.9	20.9	25.3
8: 5	101	12.0	13.0	14.2	15.8	18.0	20.9	25.5
8: 6	102	12.0	13.0	14.3	15.9	18.0	21.0	25.6
8: 7	103	12.0	13.0	14.3	15.9	18.1	21.1	25.8
8: 8	104	12.0	13.0	14.3	15.9	18.1	21.2	25.9
8: 9	105	12.0	13.1	14.3	16.0	18.2	21.3	26.1
8: 10	106	12.1	13.1	14.4	16.0	18.2	21.3	26.2
8: 11	107	12.1	13.1	14.4	16.1	18.3	21.4	26.4
9: 0	108	12.1	13.1	14.4	16.1	18.3	21.5	26.5
9: 1	109	12.1	13.2	14.5	16.1	18.4	21.6	26.7
9: 2	110	12.1	13.2	14.5	16.2	18.4	21.7	26.8
9: 3	111	12.2	13.2	14.5	16.2	18.5	21.8	27.0
9: 4	112	12.2	13.2	14.6	16.3	18.6	21.9	27.2
9: 5	113	12.2	13.3	14.6	16.3	18.6	21.9	27.3
9: 6	114	12.2	13.3	14.6	16.3	18.7	22.0	27.5
9: 7	115	12.3	13.3	14.7	16.4	18.7	22.1	27.6
9: 8	116	12.3	13.4	14.7	16.4	18.8	22.2	27.8
9: 9	117	12.3	13.4	14.7	16.5	18.8	22.3	27.9
9: 10	118	12.3	13.4	14.8	16.5	18.9	22.4	28.1
9: 11	119	12.4	13.4	14.8	16.6	19.0	22.5	28.2
10: 0	120	12.4	13.5	14.8	16.6	19.0	22.6	28.4

BMI-for-age GIRLS
5 to 19 years (z-scores)

World Health Organization

Year: Month	Months	-3 SD	-2 SD	-1 SD	Median	1 SD	2 SD	3 SD
10: 1	121	12.4	13.5	14.9	16.7	19.1	22.7	28.5
10: 2	122	12.4	13.5	14.9	16.7	19.2	22.8	28.7
10: 3	123	12.5	13.6	15.0	16.8	19.2	22.8	28.8
10: 4	124	12.5	13.6	15.0	16.8	19.3	22.9	29.0
10: 5	125	12.5	13.6	15.0	16.9	19.4	23.0	29.1
10: 6	126	12.5	13.7	15.1	16.9	19.4	23.1	29.3
10: 7	127	12.6	13.7	15.1	17.0	19.5	23.2	29.4
10: 8	128	12.6	13.7	15.2	17.0	19.6	23.3	29.6
10: 9	129	12.6	13.8	15.2	17.1	19.6	23.4	29.7
10: 10	130	12.7	13.8	15.3	17.1	19.7	23.5	29.9
10: 11	131	12.7	13.8	15.3	17.2	19.8	23.6	30.0
11: 0	132	12.7	13.9	15.3	17.2	19.9	23.7	30.2
11: 1	133	12.8	13.9	15.4	17.3	19.9	23.8	30.3
11: 2	134	12.8	14.0	15.4	17.4	20.0	23.9	30.5
11: 3	135	12.8	14.0	15.5	17.4	20.1	24.0	30.6
11: 4	136	12.9	14.0	15.5	17.5	20.2	24.1	30.8
11: 5	137	12.9	14.1	15.6	17.5	20.2	24.2	30.9
11: 6	138	12.9	14.1	15.6	17.6	20.3	24.3	31.1
11: 7	139	13.0	14.2	15.7	17.7	20.4	24.4	31.2
11: 8	140	13.0	14.2	15.7	17.7	20.5	24.5	31.4
11: 9	141	13.0	14.3	15.8	17.8	20.6	24.7	31.5
11: 10	142	13.1	14.3	15.8	17.9	20.6	24.8	31.6
11: 11	143	13.1	14.3	15.9	17.9	20.7	24.9	31.8
12: 0	144	13.2	14.4	16.0	18.0	20.8	25.0	31.9
12: 1	145	13.2	14.4	16.0	18.1	20.9	25.1	32.0
12: 2	146	13.2	14.5	16.1	18.1	21.0	25.2	32.2
12: 3	147	13.3	14.5	16.1	18.2	21.1	25.3	32.3
12: 4	148	13.3	14.6	16.2	18.3	21.1	25.4	32.4
12: 5	149	13.3	14.6	16.2	18.3	21.2	25.5	32.6
12: 6	150	13.4	14.7	16.3	18.4	21.3	25.6	32.7

BMI-for-age GIRLS
5 to 19 years (z-scores)

World Health Organization

Year: Month	Months	-3 SD	-2 SD	-1 SD	Median	1 SD	2 SD	3 SD
12: 7	151	13.4	14.7	16.3	18.5	21.4	25.7	32.8
12: 8	152	13.5	14.8	16.4	18.5	21.5	25.8	33.0
12: 9	153	13.5	14.8	16.4	18.6	21.6	25.9	33.1
12: 10	154	13.5	14.8	16.5	18.7	21.6	26.0	33.2
12: 11	155	13.6	14.9	16.6	18.7	21.7	26.1	33.3
13: 0	156	13.6	14.9	16.6	18.8	21.8	26.2	33.4
13: 1	157	13.6	15.0	16.7	18.9	21.9	26.3	33.6
13: 2	158	13.7	15.0	16.7	18.9	22.0	26.4	33.7
13: 3	159	13.7	15.1	16.8	19.0	22.0	26.5	33.8
13: 4	160	13.8	15.1	16.8	19.1	22.1	26.6	33.9
13: 5	161	13.8	15.2	16.9	19.1	22.2	26.7	34.0
13: 6	162	13.8	15.2	16.9	19.2	22.3	26.8	34.1
13: 7	163	13.9	15.2	17.0	19.3	22.4	26.9	34.2
13: 8	164	13.9	15.3	17.0	19.3	22.4	27.0	34.3
13: 9	165	13.9	15.3	17.1	19.4	22.5	27.1	34.4
13: 10	166	14.0	15.4	17.1	19.4	22.6	27.1	34.5
13: 11	167	14.0	15.4	17.2	19.5	22.7	27.2	34.6
14: 0	168	14.0	15.4	17.2	19.6	22.7	27.3	34.7
14: 1	169	14.1	15.5	17.3	19.6	22.8	27.4	34.7
14: 2	170	14.1	15.5	17.3	19.7	22.9	27.5	34.8
14: 3	171	14.1	15.6	17.4	19.7	22.9	27.6	34.9
14: 4	172	14.1	15.6	17.4	19.8	23.0	27.7	35.0
14: 5	173	14.2	15.6	17.5	19.9	23.1	27.7	35.1
14: 6	174	14.2	15.7	17.5	19.9	23.1	27.8	35.1
14: 7	175	14.2	15.7	17.6	20.0	23.2	27.9	35.2
14: 8	176	14.3	15.7	17.6	20.0	23.3	28.0	35.3
14: 9	177	14.3	15.8	17.6	20.1	23.3	28.0	35.4
14: 10	178	14.3	15.8	17.7	20.1	23.4	28.1	35.4
14: 11	179	14.3	15.8	17.7	20.2	23.5	28.2	35.5
15: 0	180	14.4	15.9	17.8	20.2	23.5	28.2	35.5

BMI-for-age GIRLS
5 to 19 years (z-scores)

World Health Organization

Year: Month	Months	-3 SD	-2 SD	-1 SD	Median	1 SD	2 SD	3 SD
15: 1	181	14.4	15.9	17.8	20.3	23.6	28.3	35.6
15: 2	182	14.4	15.9	17.8	20.3	23.6	28.4	35.7
15: 3	183	14.4	16.0	17.9	20.4	23.7	28.4	35.7
15: 4	184	14.5	16.0	17.9	20.4	23.7	28.5	35.8
15: 5	185	14.5	16.0	17.9	20.4	23.8	28.5	35.8
15: 6	186	14.5	16.0	18.0	20.5	23.8	28.6	35.8
15: 7	187	14.5	16.1	18.0	20.5	23.9	28.6	35.9
15: 8	188	14.5	16.1	18.0	20.6	23.9	28.7	35.9
15: 9	189	14.5	16.1	18.1	20.6	24.0	28.7	36.0
15: 10	190	14.6	16.1	18.1	20.6	24.0	28.8	36.0
15: 11	191	14.6	16.2	18.1	20.7	24.1	28.8	36.0
16: 0	192	14.6	16.2	18.2	20.7	24.1	28.9	36.1
16: 1	193	14.6	16.2	18.2	20.7	24.1	28.9	36.1
16: 2	194	14.6	16.2	18.2	20.8	24.2	29.0	36.1
16: 3	195	14.6	16.2	18.2	20.8	24.2	29.0	36.1
16: 4	196	14.6	16.2	18.3	20.8	24.3	29.0	36.2
16: 5	197	14.6	16.3	18.3	20.9	24.3	29.1	36.2
16: 6	198	14.7	16.3	18.3	20.9	24.3	29.1	36.2
16: 7	199	14.7	16.3	18.3	20.9	24.4	29.1	36.2
16: 8	200	14.7	16.3	18.3	20.9	24.4	29.2	36.2
16: 9	201	14.7	16.3	18.4	21.0	24.4	29.2	36.3
16: 10	202	14.7	16.3	18.4	21.0	24.4	29.2	36.3
16: 11	203	14.7	16.3	18.4	21.0	24.5	29.3	36.3
17: 0	204	14.7	16.4	18.4	21.0	24.5	29.3	36.3
17: 1	205	14.7	16.4	18.4	21.1	24.5	29.3	36.3
17: 2	206	14.7	16.4	18.4	21.1	24.6	29.3	36.3
17: 3	207	14.7	16.4	18.5	21.1	24.6	29.4	36.3
17: 4	208	14.7	16.4	18.5	21.1	24.6	29.4	36.3
17: 5	209	14.7	16.4	18.5	21.1	24.6	29.4	36.3
17: 6	210	14.7	16.4	18.5	21.2	24.6	29.4	36.3

BMI-for-age GIRLS
5 to 19 years (z-scores)

World Health Organization

Year: Month	Months	-3 SD	-2 SD	-1 SD	Median	1 SD	2 SD	3 SD
17: 7	211	14.7	16.4	18.5	21.2	24.7	29.4	36.3
17: 8	212	14.7	16.4	18.5	21.2	24.7	29.5	36.3
17: 9	213	14.7	16.4	18.5	21.2	24.7	29.5	36.3
17: 10	214	14.7	16.4	18.5	21.2	24.7	29.5	36.3
17: 11	215	14.7	16.4	18.6	21.2	24.8	29.5	36.3
18: 0	216	14.7	16.4	18.6	21.3	24.8	29.5	36.3
18: 1	217	14.7	16.5	18.6	21.3	24.8	29.5	36.3
18: 2	218	14.7	16.5	18.6	21.3	24.8	29.6	36.3
18: 3	219	14.7	16.5	18.6	21.3	24.8	29.6	36.3
18: 4	220	14.7	16.5	18.6	21.3	24.8	29.6	36.3
18: 5	221	14.7	16.5	18.6	21.3	24.9	29.6	36.2
18: 6	222	14.7	16.5	18.6	21.3	24.9	29.6	36.2
18: 7	223	14.7	16.5	18.6	21.4	24.9	29.6	36.2
18: 8	224	14.7	16.5	18.6	21.4	24.9	29.6	36.2
18: 9	225	14.7	16.5	18.7	21.4	24.9	29.6	36.2
18: 10	226	14.7	16.5	18.7	21.4	24.9	29.6	36.2
18: 11	227	14.7	16.5	18.7	21.4	25.0	29.7	36.2
19: 0	228	14.7	16.5	18.7	21.4	25.0	29.7	36.2

2007 WHO Reference

Simplified field tables

BMI-for-age BOYS
5 to 19 years (z-scores)

World Health Organization

Year: Month	Months	-3 SD	-2 SD	-1 SD	Median	1 SD	2 SD	3 SD
5: 1	61	12.1	13.0	14.1	15.3	16.6	18.3	20.2
5: 2	62	12.1	13.0	14.1	15.3	16.6	18.3	20.2
5: 3	63	12.1	13.0	14.1	15.3	16.7	18.3	20.2
5: 4	64	12.1	13.0	14.1	15.3	16.7	18.3	20.3
5: 5	65	12.1	13.0	14.1	15.3	16.7	18.3	20.3
5: 6	66	12.1	13.0	14.1	15.3	16.7	18.4	20.4
5: 7	67	12.1	13.0	14.1	15.3	16.7	18.4	20.4
5: 8	68	12.1	13.0	14.1	15.3	16.7	18.4	20.5
5: 9	69	12.1	13.0	14.1	15.3	16.7	18.4	20.5
5: 10	70	12.1	13.0	14.1	15.3	16.7	18.5	20.6
5: 11	71	12.1	13.0	14.1	15.3	16.7	18.5	20.6
6: 0	72	12.1	13.0	14.1	15.3	16.8	18.5	20.7
6: 1	73	12.1	13.0	14.1	15.3	16.8	18.6	20.8
6: 2	74	12.2	13.1	14.1	15.3	16.8	18.6	20.8
6: 3	75	12.2	13.1	14.1	15.3	16.8	18.6	20.9
6: 4	76	12.2	13.1	14.1	15.4	16.8	18.7	21.0
6: 5	77	12.2	13.1	14.1	15.4	16.9	18.7	21.0
6: 6	78	12.2	13.1	14.1	15.4	16.9	18.7	21.1
6: 7	79	12.2	13.1	14.1	15.4	16.9	18.8	21.2
6: 8	80	12.2	13.1	14.2	15.4	16.9	18.8	21.3
6: 9	81	12.2	13.1	14.2	15.4	17.0	18.9	21.3
6: 10	82	12.2	13.1	14.2	15.4	17.0	18.9	21.4
6: 11	83	12.2	13.1	14.2	15.5	17.0	19.0	21.5
7: 0	84	12.3	13.1	14.2	15.5	17.0	19.0	21.6
7: 1	85	12.3	13.2	14.2	15.5	17.1	19.1	21.7
7: 2	86	12.3	13.2	14.2	15.5	17.1	19.1	21.8
7: 3	87	12.3	13.2	14.3	15.5	17.1	19.2	21.9
7: 4	88	12.3	13.2	14.3	15.6	17.2	19.2	22.0
7: 5	89	12.3	13.2	14.3	15.6	17.2	19.3	22.0
7: 6	90	12.3	13.2	14.3	15.6	17.2	19.3	22.1

BMI-for-age BOYS
5 to 19 years (z-scores)

Year: Month	Months	-3 SD	-2 SD	-1 SD	Median	1 SD	2 SD	3 SD
7: 7	91	12.3	13.2	14.3	15.6	17.3	19.4	22.2
7: 8	92	12.3	13.2	14.3	15.6	17.3	19.4	22.4
7: 9	93	12.4	13.3	14.3	15.7	17.3	19.5	22.5
7: 10	94	12.4	13.3	14.4	15.7	17.4	19.6	22.6
7: 11	95	12.4	13.3	14.4	15.7	17.4	19.6	22.7
8: 0	96	12.4	13.3	14.4	15.7	17.4	19.7	22.8
8: 1	97	12.4	13.3	14.4	15.8	17.5	19.7	22.9
8: 2	98	12.4	13.3	14.4	15.8	17.5	19.8	23.0
8: 3	99	12.4	13.3	14.4	15.8	17.5	19.9	23.1
8: 4	100	12.4	13.4	14.5	15.8	17.6	19.9	23.3
8: 5	101	12.5	13.4	14.5	15.9	17.6	20.0	23.4
8: 6	102	12.5	13.4	14.5	15.9	17.7	20.1	23.5
8: 7	103	12.5	13.4	14.5	15.9	17.7	20.1	23.6
8: 8	104	12.5	13.4	14.5	15.9	17.7	20.2	23.8
8: 9	105	12.5	13.4	14.6	16.0	17.8	20.3	23.9
8: 10	106	12.5	13.5	14.6	16.0	17.8	20.3	24.0
8: 11	107	12.5	13.5	14.6	16.0	17.9	20.4	24.2
9: 0	108	12.6	13.5	14.6	16.0	17.9	20.5	24.3
9: 1	109	12.6	13.5	14.6	16.1	18.0	20.5	24.4
9: 2	110	12.6	13.5	14.7	16.1	18.0	20.6	24.6
9: 3	111	12.6	13.5	14.7	16.1	18.0	20.7	24.7
9: 4	112	12.6	13.6	14.7	16.2	18.1	20.8	24.9
9: 5	113	12.6	13.6	14.7	16.2	18.1	20.8	25.0
9: 6	114	12.7	13.6	14.8	16.2	18.2	20.9	25.1
9: 7	115	12.7	13.6	14.8	16.3	18.2	21.0	25.3
9: 8	116	12.7	13.6	14.8	16.3	18.3	21.1	25.5
9: 9	117	12.7	13.7	14.8	16.3	18.3	21.2	25.6
9: 10	118	12.7	13.7	14.9	16.4	18.4	21.2	25.8
9: 11	119	12.8	13.7	14.9	16.4	18.4	21.3	25.9
10: 0	120	12.8	13.7	14.9	16.4	18.5	21.4	26.1

BMI-for-age BOYS
5 to 19 years (z-scores)

World Health Organization

Year: Month	Months	-3 SD	-2 SD	-1 SD	Median	1 SD	2 SD	3 SD
10: 1	121	12.8	13.8	15.0	16.5	18.5	21.5	26.2
10: 2	122	12.8	13.8	15.0	16.5	18.6	21.6	26.4
10: 3	123	12.8	13.8	15.0	16.6	18.6	21.7	26.6
10: 4	124	12.9	13.8	15.0	16.6	18.7	21.7	26.7
10: 5	125	12.9	13.9	15.1	16.6	18.8	21.8	26.9
10: 6	126	12.9	13.9	15.1	16.7	18.8	21.9	27.0
10: 7	127	12.9	13.9	15.1	16.7	18.9	22.0	27.2
10: 8	128	13.0	13.9	15.2	16.8	18.9	22.1	27.4
10: 9	129	13.0	14.0	15.2	16.8	19.0	22.2	27.5
10: 10	130	13.0	14.0	15.2	16.9	19.0	22.3	27.7
10: 11	131	13.0	14.0	15.3	16.9	19.1	22.4	27.9
11: 0	132	13.1	14.1	15.3	16.9	19.2	22.5	28.0
11: 1	133	13.1	14.1	15.3	17.0	19.2	22.5	28.2
11: 2	134	13.1	14.1	15.4	17.0	19.3	22.6	28.4
11: 3	135	13.1	14.1	15.4	17.1	19.3	22.7	28.5
11: 4	136	13.2	14.2	15.5	17.1	19.4	22.8	28.7
11: 5	137	13.2	14.2	15.5	17.2	19.5	22.9	28.8
11: 6	138	13.2	14.2	15.5	17.2	19.5	23.0	29.0
11: 7	139	13.2	14.3	15.6	17.3	19.6	23.1	29.2
11: 8	140	13.3	14.3	15.6	17.3	19.7	23.2	29.3
11: 9	141	13.3	14.3	15.7	17.4	19.7	23.3	29.5
11: 10	142	13.3	14.4	15.7	17.4	19.8	23.4	29.6
11: 11	143	13.4	14.4	15.7	17.5	19.9	23.5	29.8
12: 0	144	13.4	14.5	15.8	17.5	19.9	23.6	30.0
12: 1	145	13.4	14.5	15.8	17.6	20.0	23.7	30.1
12: 2	146	13.5	14.5	15.9	17.6	20.1	23.8	30.3
12: 3	147	13.5	14.6	15.9	17.7	20.2	23.9	30.4
12: 4	148	13.5	14.6	16.0	17.8	20.2	24.0	30.6
12: 5	149	13.6	14.6	16.0	17.8	20.3	24.1	30.7
12: 6	150	13.6	14.7	16.1	17.9	20.4	24.2	30.9

BMI-for-age BOYS
5 to 19 years (z-scores)

World Health Organization

Year: Month	Months	-3 SD	-2 SD	-1 SD	Median	1 SD	2 SD	3 SD
12: 7	151	13.6	14.7	16.1	17.9	20.4	24.3	31.0
12: 8	152	13.7	14.8	16.2	18.0	20.5	24.4	31.1
12: 9	153	13.7	14.8	16.2	18.0	20.6	24.5	31.3
12: 10	154	13.7	14.8	16.3	18.1	20.7	24.6	31.4
12: 11	155	13.8	14.9	16.3	18.2	20.8	24.7	31.6
13: 0	156	13.8	14.9	16.4	18.2	20.8	24.8	31.7
13: 1	157	13.8	15.0	16.4	18.3	20.9	24.9	31.8
13: 2	158	13.9	15.0	16.5	18.4	21.0	25.0	31.9
13: 3	159	13.9	15.1	16.5	18.4	21.1	25.1	32.1
13: 4	160	14.0	15.1	16.6	18.5	21.1	25.2	32.2
13: 5	161	14.0	15.2	16.6	18.6	21.2	25.2	32.3
13: 6	162	14.0	15.2	16.7	18.6	21.3	25.3	32.4
13: 7	163	14.1	15.2	16.7	18.7	21.4	25.4	32.6
13: 8	164	14.1	15.3	16.8	18.7	21.5	25.5	32.7
13: 9	165	14.1	15.3	16.8	18.8	21.5	25.6	32.8
13: 10	166	14.2	15.4	16.9	18.9	21.6	25.7	32.9
13: 11	167	14.2	15.4	17.0	18.9	21.7	25.8	33.0
14: 0	168	14.3	15.5	17.0	19.0	21.8	25.9	33.1
14: 1	169	14.3	15.5	17.1	19.1	21.8	26.0	33.2
14: 2	170	14.3	15.6	17.1	19.1	21.9	26.1	33.3
14: 3	171	14.4	15.6	17.2	19.2	22.0	26.2	33.4
14: 4	172	14.4	15.7	17.2	19.3	22.1	26.3	33.5
14: 5	173	14.5	15.7	17.3	19.3	22.2	26.4	33.5
14: 6	174	14.5	15.7	17.3	19.4	22.2	26.5	33.6
14: 7	175	14.5	15.8	17.4	19.5	22.3	26.5	33.7
14: 8	176	14.6	15.8	17.4	19.5	22.4	26.6	33.8
14: 9	177	14.6	15.9	17.5	19.6	22.5	26.7	33.9
14: 10	178	14.6	15.9	17.5	19.6	22.5	26.8	33.9
14: 11	179	14.7	16.0	17.6	19.7	22.6	26.9	34.0
15: 0	180	14.7	16.0	17.6	19.8	22.7	27.0	34.1

BMI-for-age BOYS
5 to 19 years (z-scores)

World Health Organization

Year: Month	Months	-3 SD	-2 SD	-1 SD	Median	1 SD	2 SD	3 SD
15: 1	181	14.7	16.1	17.7	19.8	22.8	27.1	34.1
15: 2	182	14.8	16.1	17.8	19.9	22.8	27.1	34.2
15: 3	183	14.8	16.1	17.8	20.0	22.9	27.2	34.3
15: 4	184	14.8	16.2	17.9	20.0	23.0	27.3	34.3
15: 5	185	14.9	16.2	17.9	20.1	23.0	27.4	34.4
15: 6	186	14.9	16.3	18.0	20.1	23.1	27.4	34.5
15: 7	187	15.0	16.3	18.0	20.2	23.2	27.5	34.5
15: 8	188	15.0	16.3	18.1	20.3	23.3	27.6	34.6
15: 9	189	15.0	16.4	18.1	20.3	23.3	27.7	34.6
15: 10	190	15.0	16.4	18.2	20.4	23.4	27.7	34.7
15: 11	191	15.1	16.5	18.2	20.4	23.5	27.8	34.7
16: 0	192	15.1	16.5	18.2	20.5	23.5	27.9	34.8
16: 1	193	15.1	16.5	18.3	20.6	23.6	27.9	34.8
16: 2	194	15.2	16.6	18.3	20.6	23.7	28.0	34.8
16: 3	195	15.2	16.6	18.4	20.7	23.7	28.1	34.9
16: 4	196	15.2	16.7	18.4	20.7	23.8	28.1	34.9
16: 5	197	15.3	16.7	18.5	20.8	23.8	28.2	35.0
16: 6	198	15.3	16.7	18.5	20.8	23.9	28.3	35.0
16: 7	199	15.3	16.8	18.6	20.9	24.0	28.3	35.0
16: 8	200	15.3	16.8	18.6	20.9	24.0	28.4	35.1
16: 9	201	15.4	16.8	18.7	21.0	24.1	28.5	35.1
16: 10	202	15.4	16.9	18.7	21.0	24.2	28.5	35.1
16: 11	203	15.4	16.9	18.7	21.1	24.2	28.6	35.2
17: 0	204	15.4	16.9	18.8	21.1	24.3	28.6	35.2
17: 1	205	15.5	17.0	18.8	21.2	24.3	28.7	35.2
17: 2	206	15.5	17.0	18.9	21.2	24.4	28.7	35.2
17: 3	207	15.5	17.0	18.9	21.3	24.4	28.8	35.3
17: 4	208	15.5	17.1	18.9	21.3	24.5	28.9	35.3
17: 5	209	15.6	17.1	19.0	21.4	24.5	28.9	35.3
17: 6	210	15.6	17.1	19.0	21.4	24.6	29.0	35.3

BMI-for-age BOYS
5 to 19 years (z-scores)

World Health Organization

Year: Month	Months	-3 SD	-2 SD	-1 SD	Median	1 SD	2 SD	3 SD
17: 7	211	15.6	17.1	19.1	21.5	24.7	29.0	35.4
17: 8	212	15.6	17.2	19.1	21.5	24.7	29.1	35.4
17: 9	213	15.6	17.2	19.1	21.6	24.8	29.1	35.4
17: 10	214	15.7	17.2	19.2	21.6	24.8	29.2	35.4
17: 11	215	15.7	17.3	19.2	21.7	24.9	29.2	35.4
18: 0	216	15.7	17.3	19.2	21.7	24.9	29.2	35.4
18: 1	217	15.7	17.3	19.3	21.8	25.0	29.3	35.4
18: 2	218	15.7	17.3	19.3	21.8	25.0	29.3	35.5
18: 3	219	15.7	17.4	19.3	21.8	25.1	29.4	35.5
18: 4	220	15.8	17.4	19.4	21.9	25.1	29.4	35.5
18: 5	221	15.8	17.4	19.4	21.9	25.1	29.5	35.5
18: 6	222	15.8	17.4	19.4	22.0	25.2	29.5	35.5
18: 7	223	15.8	17.5	19.5	22.0	25.2	29.5	35.5
18: 8	224	15.8	17.5	19.5	22.0	25.3	29.6	35.5
18: 9	225	15.8	17.5	19.5	22.1	25.3	29.6	35.5
18: 10	226	15.8	17.5	19.6	22.1	25.4	29.6	35.5
18: 11	227	15.8	17.5	19.6	22.2	25.4	29.7	35.5
19: 0	228	15.9	17.6	19.6	22.2	25.4	29.7	35.5

2007 WHO Reference

Simplified field tables

Weight-for-height GIRLS
2 to 5 years (z-scores)

World Health Organization

cm	-3 SD	-2 SD	-1 SD	Median	1 SD	2 SD	3 SD
65.0	5.6	6.1	6.6	7.2	7.9	8.7	9.7
65.5	5.7	6.2	6.7	7.4	8.1	8.9	9.8
66.0	5.8	6.3	6.8	7.5	8.2	9.0	10.0
66.5	5.8	6.4	6.9	7.6	8.3	9.1	10.1
67.0	5.9	6.4	7.0	7.7	8.4	9.3	10.2
67.5	6.0	6.5	7.1	7.8	8.5	9.4	10.4
68.0	6.1	6.6	7.2	7.9	8.7	9.5	10.5
68.5	6.2	6.7	7.3	8.0	8.8	9.7	10.7
69.0	6.3	6.8	7.4	8.1	8.9	9.8	10.8
69.5	6.3	6.9	7.5	8.2	9.0	9.9	10.9
70.0	6.4	7.0	7.6	8.3	9.1	10.0	11.1
70.5	6.5	7.1	7.7	8.4	9.2	10.1	11.2
71.0	6.6	7.1	7.8	8.5	9.3	10.3	11.3
71.5	6.7	7.2	7.9	8.6	9.4	10.4	11.5
72.0	6.7	7.3	8.0	8.7	9.5	10.5	11.6
72.5	6.8	7.4	8.1	8.8	9.7	10.6	11.7
73.0	6.9	7.5	8.1	8.9	9.8	10.7	11.8
73.5	7.0	7.6	8.2	9.0	9.9	10.8	12.0
74.0	7.0	7.6	8.3	9.1	10.0	11.0	12.1
74.5	7.1	7.7	8.4	9.2	10.1	11.1	12.2
75.0	7.2	7.8	8.5	9.3	10.2	11.2	12.3
75.5	7.2	7.9	8.6	9.4	10.3	11.3	12.5
76.0	7.3	8.0	8.7	9.5	10.4	11.4	12.6
76.5	7.4	8.0	8.7	9.6	10.5	11.5	12.7
77.0	7.5	8.1	8.8	9.6	10.6	11.6	12.8
77.5	7.5	8.2	8.9	9.7	10.7	11.7	12.9
78.0	7.6	8.3	9.0	9.8	10.8	11.8	13.1
78.5	7.7	8.4	9.1	9.9	10.9	12.0	13.2
79.0	7.8	8.4	9.2	10.0	11.0	12.1	13.3
79.5	7.8	8.5	9.3	10.1	11.1	12.2	13.4

Weight-for-height GIRLS
2 to 5 years (z-scores)

World Health Organization

cm	-3 SD	-2 SD	-1 SD	Median	1 SD	2 SD	3 SD
80.0	7.9	8.6	9.4	10.2	11.2	12.3	13.6
80.5	8.0	8.7	9.5	10.3	11.3	12.4	13.7
81.0	8.1	8.8	9.6	10.4	11.4	12.6	13.9
81.5	8.2	8.9	9.7	10.6	11.6	12.7	14.0
82.0	8.3	9.0	9.8	10.7	11.7	12.8	14.1
82.5	8.4	9.1	9.9	10.8	11.8	13.0	14.3
83.0	8.5	9.2	10.0	10.9	11.9	13.1	14.5
83.5	8.5	9.3	10.1	11.0	12.1	13.3	14.6
84.0	8.6	9.4	10.2	11.1	12.2	13.4	14.8
84.5	8.7	9.5	10.3	11.3	12.3	13.5	14.9
85.0	8.8	9.6	10.4	11.4	12.5	13.7	15.1
85.5	8.9	9.7	10.6	11.5	12.6	13.8	15.3
86.0	9.0	9.8	10.7	11.6	12.7	14.0	15.4
86.5	9.1	9.9	10.8	11.8	12.9	14.2	15.6
87.0	9.2	10.0	10.9	11.9	13.0	14.3	15.8
87.5	9.3	10.1	11.0	12.0	13.2	14.5	15.9
88.0	9.4	10.2	11.1	12.1	13.3	14.6	16.1
88.5	9.5	10.3	11.2	12.3	13.4	14.8	16.3
89.0	9.6	10.4	11.4	12.4	13.6	14.9	16.4
89.5	9.7	10.5	11.5	12.5	13.7	15.1	16.6
90.0	9.8	10.6	11.6	12.6	13.8	15.2	16.8
90.5	9.9	10.7	11.7	12.8	14.0	15.4	16.9
91.0	10.0	10.9	11.8	12.9	14.1	15.5	17.1
91.5	10.1	11.0	11.9	13.0	14.3	15.7	17.3
92.0	10.2	11.1	12.0	13.1	14.4	15.8	17.4
92.5	10.3	11.2	12.1	13.3	14.5	16.0	17.6
93.0	10.4	11.3	12.3	13.4	14.7	16.1	17.8
93.5	10.5	11.4	12.4	13.5	14.8	16.3	17.9
94.0	10.6	11.5	12.5	13.6	14.9	16.4	18.1
94.5	10.7	11.6	12.6	13.8	15.1	16.6	18.3
95.0	10.8	11.7	12.7	13.9	15.2	16.7	18.5

Weight-for-height GIRLS
2 to 5 years (z-scores)

World Health Organization

cm	-3 SD	-2 SD	-1 SD	Median	1 SD	2 SD	3 SD
95.5	10.8	11.8	12.8	14.0	15.4	16.9	18.6
96.0	10.9	11.9	12.9	14.1	15.5	17.0	18.8
96.5	11.0	12.0	13.1	14.3	15.6	17.2	19.0
97.0	11.1	12.1	13.2	14.4	15.8	17.4	19.2
97.5	11.2	12.2	13.3	14.5	15.9	17.5	19.3
98.0	11.3	12.3	13.4	14.7	16.1	17.7	19.5
98.5	11.4	12.4	13.5	14.8	16.2	17.9	19.7
99.0	11.5	12.5	13.7	14.9	16.4	18.0	19.9
99.5	11.6	12.7	13.8	15.1	16.5	18.2	20.1
100.0	11.7	12.8	13.9	15.2	16.7	18.4	20.3
100.5	11.9	12.9	14.1	15.4	16.9	18.6	20.5
101.0	12.0	13.0	14.2	15.5	17.0	18.7	20.7
101.5	12.1	13.1	14.3	15.7	17.2	18.9	20.9
102.0	12.2	13.3	14.5	15.8	17.4	19.1	21.1
102.5	12.3	13.4	14.6	16.0	17.5	19.3	21.4
103.0	12.4	13.5	14.7	16.1	17.7	19.5	21.6
103.5	12.5	13.6	14.9	16.3	17.9	19.7	21.8
104.0	12.6	13.8	15.0	16.4	18.1	19.9	22.0
104.5	12.8	13.9	15.2	16.6	18.2	20.1	22.3
105.0	12.9	14.0	15.3	16.8	18.4	20.3	22.5
105.5	13.0	14.2	15.5	16.9	18.6	20.5	22.7
106.0	13.1	14.3	15.6	17.1	18.8	20.8	23.0
106.5	13.3	14.5	15.8	17.3	19.0	21.0	23.2
107.0	13.4	14.6	15.9	17.5	19.2	21.2	23.5
107.5	13.5	14.7	16.1	17.7	19.4	21.4	23.7
108.0	13.7	14.9	16.3	17.8	19.6	21.7	24.0
108.5	13.8	15.0	16.4	18.0	19.8	21.9	24.3
109.0	13.9	15.2	16.6	18.2	20.0	22.1	24.5
109.5	14.1	15.4	16.8	18.4	20.3	22.4	24.8
110.0	14.2	15.5	17.0	18.6	20.5	22.6	25.1
110.5	14.4	15.7	17.1	18.8	20.7	22.9	25.4

Weight-for-height GIRLS
2 to 5 years (z-scores)

World Health Organization

cm	-3 SD	-2 SD	-1 SD	Median	1 SD	2 SD	3 SD
111.0	14.5	15.8	17.3	19.0	20.9	23.1	25.7
111.5	14.7	16.0	17.5	19.2	21.2	23.4	26.0
112.0	14.8	16.2	17.7	19.4	21.4	23.6	26.2
112.5	15.0	16.3	17.9	19.6	21.6	23.9	26.5
113.0	15.1	16.5	18.0	19.8	21.8	24.2	26.8
113.5	15.3	16.7	18.2	20.0	22.1	24.4	27.1
114.0	15.4	16.8	18.4	20.2	22.3	24.7	27.4
114.5	15.6	17.0	18.6	20.5	22.6	25.0	27.8
115.0	15.7	17.2	18.8	20.7	22.8	25.2	28.1
115.5	15.9	17.3	19.0	20.9	23.0	25.5	28.4
116.0	16.0	17.5	19.2	21.1	23.3	25.8	28.7
116.5	16.2	17.7	19.4	21.3	23.5	26.1	29.0
117.0	16.3	17.8	19.6	21.5	23.8	26.3	29.3
117.5	16.5	18.0	19.8	21.7	24.0	26.6	29.6
118.0	16.6	18.2	19.9	22.0	24.2	26.9	29.9
118.5	16.8	18.4	20.1	22.2	24.5	27.2	30.3
119.0	16.9	18.5	20.3	22.4	24.7	27.4	30.6
119.5	17.1	18.7	20.5	22.6	25.0	27.7	30.9
120.0	17.3	18.9	20.7	22.8	25.2	28.0	31.2

WHO Child Growth Standards

Simplified field tables

Weight-for-height BOYS
2 to 5 years (z-scores)

World Health Organization

cm	-3 SD	-2 SD	-1 SD	Median	1 SD	2 SD	3 SD
65.0	5.9	6.3	6.9	7.4	8.1	8.8	9.6
65.5	6.0	6.4	7.0	7.6	8.2	8.9	9.8
66.0	6.1	6.5	7.1	7.7	8.3	9.1	9.9
66.5	6.1	6.6	7.2	7.8	8.5	9.2	10.1
67.0	6.2	6.7	7.3	7.9	8.6	9.4	10.2
67.5	6.3	6.8	7.4	8.0	8.7	9.5	10.4
68.0	6.4	6.9	7.5	8.1	8.8	9.6	10.5
68.5	6.5	7.0	7.6	8.2	9.0	9.8	10.7
69.0	6.6	7.1	7.7	8.4	9.1	9.9	10.8
69.5	6.7	7.2	7.8	8.5	9.2	10.0	11.0
70.0	6.8	7.3	7.9	8.6	9.3	10.2	11.1
70.5	6.9	7.4	8.0	8.7	9.5	10.3	11.3
71.0	6.9	7.5	8.1	8.8	9.6	10.4	11.4
71.5	7.0	7.6	8.2	8.9	9.7	10.6	11.6
72.0	7.1	7.7	8.3	9.0	9.8	10.7	11.7
72.5	7.2	7.8	8.4	9.1	9.9	10.8	11.8
73.0	7.3	7.9	8.5	9.2	10.0	11.0	12.0
73.5	7.4	7.9	8.6	9.3	10.2	11.1	12.1
74.0	7.4	8.0	8.7	9.4	10.3	11.2	12.2
74.5	7.5	8.1	8.8	9.5	10.4	11.3	12.4
75.0	7.6	8.2	8.9	9.6	10.5	11.4	12.5
75.5	7.7	8.3	9.0	9.7	10.6	11.6	12.6
76.0	7.7	8.4	9.1	9.8	10.7	11.7	12.8
76.5	7.8	8.5	9.2	9.9	10.8	11.8	12.9
77.0	7.9	8.5	9.2	10.0	10.9	11.9	13.0
77.5	8.0	8.6	9.3	10.1	11.0	12.0	13.1
78.0	8.0	8.7	9.4	10.2	11.1	12.1	13.3
78.5	8.1	8.8	9.5	10.3	11.2	12.2	13.4
79.0	8.2	8.8	9.6	10.4	11.3	12.3	13.5
79.5	8.3	8.9	9.7	10.5	11.4	12.4	13.6

Weight-for-height BOYS
2 to 5 years (z-scores)

World Health Organization

cm	-3 SD	-2 SD	-1 SD	Median	1 SD	2 SD	3 SD
80.0	8.3	9.0	9.7	10.6	11.5	12.6	13.7
80.5	8.4	9.1	9.8	10.7	11.6	12.7	13.8
81.0	8.5	9.2	9.9	10.8	11.7	12.8	14.0
81.5	8.6	9.3	10.0	10.9	11.8	12.9	14.1
82.0	8.7	9.3	10.1	11.0	11.9	13.0	14.2
82.5	8.7	9.4	10.2	11.1	12.1	13.1	14.4
83.0	8.8	9.5	10.3	11.2	12.2	13.3	14.5
83.5	8.9	9.6	10.4	11.3	12.3	13.4	14.6
84.0	9.0	9.7	10.5	11.4	12.4	13.5	14.8
84.5	9.1	9.9	10.7	11.5	12.5	13.7	14.9
85.0	9.2	10.0	10.8	11.7	12.7	13.8	15.1
85.5	9.3	10.1	10.9	11.8	12.8	13.9	15.2
86.0	9.4	10.2	11.0	11.9	12.9	14.1	15.4
86.5	9.5	10.3	11.1	12.0	13.1	14.2	15.5
87.0	9.6	10.4	11.2	12.2	13.2	14.4	15.7
87.5	9.7	10.5	11.3	12.3	13.3	14.5	15.8
88.0	9.8	10.6	11.5	12.4	13.5	14.7	16.0
88.5	9.9	10.7	11.6	12.5	13.6	14.8	16.1
89.0	10.0	10.8	11.7	12.6	13.7	14.9	16.3
89.5	10.1	10.9	11.8	12.8	13.9	15.1	16.4
90.0	10.2	11.0	11.9	12.9	14.0	15.2	16.6
90.5	10.3	11.1	12.0	13.0	14.1	15.3	16.7
91.0	10.4	11.2	12.1	13.1	14.2	15.5	16.9
91.5	10.5	11.3	12.2	13.2	14.4	15.6	17.0
92.0	10.6	11.4	12.3	13.4	14.5	15.8	17.2
92.5	10.7	11.5	12.4	13.5	14.6	15.9	17.3
93.0	10.8	11.6	12.6	13.6	14.7	16.0	17.5
93.5	10.9	11.7	12.7	13.7	14.9	16.2	17.6
94.0	11.0	11.8	12.8	13.8	15.0	16.3	17.8
94.5	11.1	11.9	12.9	13.9	15.1	16.5	17.9
95.0	11.1	12.0	13.0	14.1	15.3	16.6	18.1

Weight-for-height BOYS
2 to 5 years (z-scores)

World Health Organization

cm	-3 SD	-2 SD	-1 SD	Median	1 SD	2 SD	3 SD
95.5	11.2	12.1	13.1	14.2	15.4	16.7	18.3
96.0	11.3	12.2	13.2	14.3	15.5	16.9	18.4
96.5	11.4	12.3	13.3	14.4	15.7	17.0	18.6
97.0	11.5	12.4	13.4	14.6	15.8	17.2	18.8
97.5	11.6	12.5	13.6	14.7	15.9	17.4	18.9
98.0	11.7	12.6	13.7	14.8	16.1	17.5	19.1
98.5	11.8	12.8	13.8	14.9	16.2	17.7	19.3
99.0	11.9	12.9	13.9	15.1	16.4	17.9	19.5
99.5	12.0	13.0	14.0	15.2	16.5	18.0	19.7
100.0	12.1	13.1	14.2	15.4	16.7	18.2	19.9
100.5	12.2	13.2	14.3	15.5	16.9	18.4	20.1
101.0	12.3	13.3	14.4	15.6	17.0	18.5	20.3
101.5	12.4	13.4	14.5	15.8	17.2	18.7	20.5
102.0	12.5	13.6	14.7	15.9	17.3	18.9	20.7
102.5	12.6	13.7	14.8	16.1	17.5	19.1	20.9
103.0	12.8	13.8	14.9	16.2	17.7	19.3	21.1
103.5	12.9	13.9	15.1	16.4	17.8	19.5	21.3
104.0	13.0	14.0	15.2	16.5	18.0	19.7	21.6
104.5	13.1	14.2	15.4	16.7	18.2	19.9	21.8
105.0	13.2	14.3	15.5	16.8	18.4	20.1	22.0
105.5	13.3	14.4	15.6	17.0	18.5	20.3	22.2
106.0	13.4	14.5	15.8	17.2	18.7	20.5	22.5
106.5	13.5	14.7	15.9	17.3	18.9	20.7	22.7
107.0	13.7	14.8	16.1	17.5	19.1	20.9	22.9
107.5	13.8	14.9	16.2	17.7	19.3	21.1	23.2
108.0	13.9	15.1	16.4	17.8	19.5	21.3	23.4
108.5	14.0	15.2	16.5	18.0	19.7	21.5	23.7
109.0	14.1	15.3	16.7	18.2	19.8	21.8	23.9
109.5	14.3	15.5	16.8	18.3	20.0	22.0	24.2
110.0	14.4	15.6	17.0	18.5	20.2	22.2	24.4
110.5	14.5	15.8	17.1	18.7	20.4	22.4	24.7

Weight-for-height BOYS
2 to 5 years (z-scores)

World Health Organization

cm	-3 SD	-2 SD	-1 SD	Median	1 SD	2 SD	3 SD
111.0	14.6	15.9	17.3	18.9	20.7	22.7	25.0
111.5	14.8	16.0	17.5	19.1	20.9	22.9	25.2
112.0	14.9	16.2	17.6	19.2	21.1	23.1	25.5
112.5	15.0	16.3	17.8	19.4	21.3	23.4	25.8
113.0	15.2	16.5	18.0	19.6	21.5	23.6	26.0
113.5	15.3	16.6	18.1	19.8	21.7	23.9	26.3
114.0	15.4	16.8	18.3	20.0	21.9	24.1	26.6
114.5	15.6	16.9	18.5	20.2	22.1	24.4	26.9
115.0	15.7	17.1	18.6	20.4	22.4	24.6	27.2
115.5	15.8	17.2	18.8	20.6	22.6	24.9	27.5
116.0	16.0	17.4	19.0	20.8	22.8	25.1	27.8
116.5	16.1	17.5	19.2	21.0	23.0	25.4	28.0
117.0	16.2	17.7	19.3	21.2	23.3	25.6	28.3
117.5	16.4	17.9	19.5	21.4	23.5	25.9	28.6
118.0	16.5	18.0	19.7	21.6	23.7	26.1	28.9
118.5	16.7	18.2	19.9	21.8	23.9	26.4	29.2
119.0	16.8	18.3	20.0	22.0	24.1	26.6	29.5
119.5	16.9	18.5	20.2	22.2	24.4	26.9	29.8
120.0	17.1	18.6	20.4	22.4	24.6	27.2	30.1

WHO Child Growth Standards
